# RbAp46/48^LIN-53^ and HAT-1 are required for initial CENP-A^HCP-3^ deposition and *de novo* holocentromere formation on artificial chromosomes in *Caenorhabditis elegans* embryos

**DOI:** 10.1093/nar/gkab217

**Published:** 2021-04-19

**Authors:** Zhongyang Lin, Karen Wing Yee Yuen

**Affiliations:** School of Biological Sciences, The University of Hong Kong. Kadoorie Biological Sciences Building, Pokfulam Road, Hong Kong; School of Biological Sciences, The University of Hong Kong. Kadoorie Biological Sciences Building, Pokfulam Road, Hong Kong

## Abstract

Foreign DNA microinjected into the *Caenorhabditis elegans* syncytial gonad forms episomal extra-chromosomal arrays, or artificial chromosomes (ACs), in embryos. Short, linear DNA fragments injected concatemerize into high molecular weight (HMW) DNA arrays that are visible as punctate DAPI-stained foci in oocytes, and they undergo chromatinization and centromerization in embryos. The inner centromere, inner kinetochore and spindle checkpoint components, including AIR-2, CENP-A^HCP-3^, Mis18BP1^KNL-2^ and BUB-1, respectively, assemble onto the nascent ACs during the first mitosis. The DNA replication efficiency of ACs improves over several cell cycles, which correlates with the improvement of kinetochore bi-orientation and proper segregation of ACs. Depletion of condensin II subunits, like CAPG-2 and SMC-4, but not the replicative helicase component, MCM-2, reduces *de novo* CENP-A^HCP-3^ level on nascent ACs. Furthermore, H3K9ac, H4K5ac and H4K12ac are highly enriched on newly chromatinized ACs. RbAp46/48^LIN-53^ and HAT-1, which affect the acetylation of histone H3 and H4, are essential for chromatinization, *de novo* centromere formation and segregation competency of nascent ACs. RbAp46/48^LIN-53^ or HAT-1 depletion causes the loss of both CENP-A^HCP-3^ and Mis18BP1^KNL-2^ initial deposition at *de novo* centromeres on ACs. This phenomenon is different from centromere maintenance on endogenous chromosomes, where Mis18BP1^KNL-2^ functions upstream of RbAp46/48^LIN-53^.

## INTRODUCTION

Histone H3 variant, CENP-A, replaces histone H3 in some of the centromeric nucleosomes and serves as the foundation for building the kinetochore (1), which connects the sister chromatids to opposite spindles and orchestrates chromosome movement in cell division. CENP-A-specific chaperone deposits CENP-A precisely to the centromeric regions of sister chromatids. Centromere propagation through cell cycles and generations is crucial for accurate chromosome segregation and maintenance of genome integrity.

Ectopic formation of a centromere can result in a dicentric chromosome, which may undergo chromosome breakage-fusion-bridge (BFB) cycle, leading to chromosomal rearrangements, chromosome losses or gains, aneuploidy, and potentially chromosome instability (CIN) and tumorigenesis. Multiple cases of neocentromeres were found in human patients with congenital abnormalities or developmental disorders (2). The mechanism of new centromere formation is still not fully understood, partly because of the technical challenges in tracing the early events of neocentromere formation in patients’ cells. However, new centromere formation has been observed in diverse species. For example, tethering CENP-A-specific chaperones to a euchromatin locus or overexpressing CENP-A could cause ectopic CENP-A localization and ectopic centromere formation in fission yeast and human cells (3,4). Besides, transforming or transfecting centromeric DNA into yeast (3,5,6) or human cells (4,7,8) can form artificial chromosomes with *de novo* centromeres. However, most artificial chromosome (AC) formation in these species often relies on the presence of their endogenous centromeric DNA sequences, which suggests endogenous centromeric DNA sequences are preferred for new centromere formation. The isolation of these ACs require long-term selection, and they have relatively low frequencies of *de novo* centromere formation, which has limited the study of the early events of new centromere formation.

In *Caenorhabditis elegans*, injecting foreign DNA, even devoid of *C. elegans* sequences, into its syncytial gonad could form episomal extra-chromosomal arrays, which we also call artificial chromosomes, in the embryonic cells. Some of these ACs acquire the ability to propagate mitotically and are also inherited through subsequent generations (9,10). These heritable ACs have established a functional holocentromere rather than hitchhiking on the endogenous chromosomes (11). Dissecting the mechanism of *de novo* centromere establishment on ACs may extend our understanding of the neocentromere formation process on endogenous chromosomes.

In the present study, after injection of short, linear DNA, we investigated the timing of *de novo* CENP-A^HCP-3^ deposition on ACs, and demonstrated that CENP-A^HCP-3^ starts to assemble on ACs after fertilization. Another inner kinetochore protein, Mis18BP1^KNL-2^, and an inner centromere protein, AIR-2, are also recruited to the nascent ACs in the first mitosis. The ACs attempt to segregate in the first cell division, but with anaphase bridges. We also analyzed the histone post-translational modifications (PTMs) that are co-present with *de novo* CENP-A^HCP-3^ on nascent ACs in one-cell embryos. Based on the profiles of the enriched histone PTMs on nascent ACs, we depleted the relevant histone modifiers or the associated histone chaperones by RNA interference (RNAi), and analyzed the AC segregation rate by live-cell imaging, and the canonical histone and centromeric protein signals by immunofluorescence analysis. We demonstrated that HAT-1 and RbAp46/48^LIN-53^ are required for the enriched H3K9ac, H4K5ac and H4K12ac on nascent ACs in one-cell embryos. Depleting HAT-1, RbAp46/48^LIN-53^ or both will reduce ACs’ segregation competency and reduce *de novo* CENP-A^HCP-3^ deposition on nascent ACs. We show that *de novo* CENP-A^HCP-3^ deposition on ACs also requires condensin II subunits, but it is independent of DNA replicative helicase component, MCM-2. These results demonstrate that the mechanism of *de novo* CENP-A^HCP-3^ deposition on ACs requires histone acetyltransferase HAT-1, and the CENP-A deposition machinery, including histone chaperone RbAp46/48^LIN-53^, together with M18BP1^KNL-2^ and condensin II.

## MATERIALS AND METHODS

### Worm strains and maintenance

Worm strains used in this study are listed in Supplementary Table S1. All worms were maintained at 22°C, unless otherwise mentioned, on MYOB plates [all components: 2.0 g NaCl, 0.55 g Tris-HCl, 0.24 g Tris-OH, 3.1 g Bacto peptone, 8 mg cholesterol, 20 g agar; mixed with 1 L water and then autoclaved] seeded with *E. coli* OP50. The CRISPR/Cas9 transgenic technique described by Dickinson and Goldstein (12) was used to design and generate a GFP-tagged HAT-1 at the endogenous locus. PCR genotyping was performed using primer set: Seq-Hat-1 (Supplementary Table S2).

### Double-stranded RNA (dsRNA) synthesis and RNA interference (RNAi)

PCR primers were designed to amplify a region of target genes from N2 *C. elegans* genomic DNA or cDNA. T3 promoter (AATTAACCCTCACTAAAGG) or T7 promoter (TAATACGACTCACTATAGG) was added to the 5′ end of primers. Primers (Supplementary Table S2) were selected using NCBI-Primer-Blast and were subjected to BLAST search using the *C. elegans* genome to confirm the primer specificity. PCR was performed using TaKaRa Ex Taq® DNA Polymerase and the PCR products were purified by Qiagen PCR purification kit. Purified PCR products were subjected to *in vitro* transcription using Ambion T3 and T7 MEGAscript® Kit at 37°C for 4–6 h. Reaction products were digested with TURBO DNase at 37°C for 15 min and purified using Ambion MEGAclear^TM^ Kit. Eluates were incubated at 68°C for 10 min followed by 37°C for 30 min for complementary RNA annealing. Annealed dsRNA was adjusted to 1 μg/μl in ddH_2_O for microinjection. For RNAi, L4 stage worms were injected with dsRNA (1 μg/μl) and recovered at 22°C for 24 h before further analysis. For RNAi plus AC introduction, L4 stage worms were injected with dsRNA (1 μg/μl) and recovered at 22°C for 18 h to reach the young adult stage. The p64xLacO plasmid DNA was linearized by AfaI and purified (known as L64xLacO). The RNAi-treated young adult worms were then injected with L64xLacO in the gonad and recovered at 22°C for another 5 h before live-cell imaging or immunofluorescence staining.

### Rapid whole worm RT-qPCR

Rapid whole worm RT-qPCR was modified from the individual worm RT-qPCR method reported previously (13). Five wild-type or RNAi-treated worms were picked into 5 μl of worm lysis buffer (5 mM Tris pH 8.0, 0.5% Triton X-100, 0.5% Tween 20, 0.25 mM EDTA and 1 mg/ml proteinase K (Thermo Fisher)), followed by 65°C incubation for 10 min, then 85°C for 1 min.

cDNA synthesis was performed using Maxima H Minus cDNA synthesis kit (Thermo Fisher). About 5 μl of cDNA synthesis mix was added to the worm lysate. The final mix contains 1× RT buffer, 0.5 mM each dNTP, 5 μM random hexamer, heat labile dsDNAse 1 unit/μl, RNAse inhibitor and 20 unit/μl reverse transcriptase. The tube was briefly centrifuged, mixed and incubated at 25°C for 10 min, followed by 55°C for 30 min and finally 85°C for 5 min. The cDNA was diluted to 100 μl with RNase-free H_2_O and 2 μl was used for each PCR reaction for a final volume of 20 μl.

Quantitative PCR was performed using StepOnePlus Real-Time PCR System using the Applied Biosystems™ Fast SYBR™ Green Master Mix with the following parameters: 95°C for 20 s and 40 cycles of 95°C for 3 s, 60°C for 30 s. All data were normalized to the *act-1* gene. The primers used are listed in Supplementary Table S1.

### Live-cell imaging and AC segregation assay

Episomal artificial chromosomes (ACs) were visualized by injecting DNA containing LacO tandem repeats into worm strain OD426 (Supplementary Table S1), as reported previously (11), except that here we used linear DNA (L64xLacO) for microinjection. Injected worms were recovered on OP50-seeded plates for 5–8 h after microinjection. About 3–4 worms were then dissected in 2 μl M9 buffer to release the embryos. Embryos were mounted on a freshly prepared 2% agarose pad, and the slide edges were sealed with Vaseline. Live-cell images were taken with a Carl Zeiss LSM710 laser scanning confocal microscope with a 16 AC Plan-Neofluar 40× Oil objective lens and PMT detectors. Stacks with 17 × 1.32 μm planes were scanned for each embryo in a 3× zoom, 1-min or 30-s time interval, with 3.15 μs pixel dwell and 92 μm pinhole. Laser power for 488 nm and 543 nm was set at 5.5% and 6.5%, respectively.

To determine the AC segregation rates, every dividing cell that contains at least one AC was counted as one sample. Each division was categorized as either containing at least a segregating AC or containing all non-segregating AC(s). Segregating ACs were defined as those that aligned with the metaphase plate and segregated with endogenous chromosomes during anaphase. Non-segregating ACs were defined as those that remained in the cytoplasm or nucleus and did not segregate in mitosis. The AC segregation rate was calculated as the number of dividing cells containing at least one segregating ACs over the total number of dividing cells containing ACs. Among the segregating ACs, some may lag and have anaphase bridges, and may not eventually equally divide.

### Immunofluorescence (IF) staining

Embryos from N2, OD426 or WYY46 (Supplementary Table S1) gravid hermaphrodites were freeze-cracked after dissection of adult worms and fixed in -20°C methanol for 30 min. Embryos were then rehydrated in PBS [137 mM NaCl, 2.7 mM KCl, 4.3 mM Na_2_HPO_4_, 1.4 mM KH_2_PO_4_] for 5 min and blocked by AbDil [4% BSA, 0.1% Triton-X 100 in PBS] at room temperature for 20 min. Primary antibody incubation, using rabbit (Rb)-anti-HCP-3 (Novus biologicals 29540002, 1:1000, animal number: Q0804; or 1:2000, animal number: G3048), Rb-anti-KNL-2 (1:500, a gift from Desai Lab), Rb-anti-SMC-4 (1:500, a gift from Desai Lab), mouse (Ms)-anti-LacI (1:250, Millipore 05–503), Rb-anti-H3K9ac (1:500; Millipore ABE18), Rb-anti-H4K5ac (1:500, Abcam ab51997), Rb-anti-H4K12ac (1:500, Abcam ab177793), Rb-anti-H3K27me3 (1:500; Millipore 07–449), Rb-anti-H3K4me1 (1:500, Abcam ab176877), Rb-anti-H3K4me2 (Novus Biologicals NB21–1022), Rb-anti-H3K4me3 (1:500, Abcam ab8580), Ms-anti-H3K9me2 (1:500 Abcam, ab1220), Rb-anti-H3K9me3 (1:500, Abcam ab8898), Rb-anti-H4K20me1 (1:1000, Abcam ab9051), Rb-anti-H3 (1:1000, Abcam ab18521) or Rb-anti-H4 (1:1000, Abcam ab10158; or 1:2000, Abcam ab177840) was performed at 4°C overnight. Slides were washed with PBST [0.1% Triton-X 100 in PBS] 3 × 10 min. The slides were then incubated with goat-anti-Ms-IgG FITC (1:500, Jackson ImmunoResearch Laboratories, 115–096-062), goat-anti-Rb-IgG Cy3-conjugated secondary antibody (1:500, Jackson ImmunoResearch Laboratories, 111–166-045) or goat-anti-Rb-IgG Alexa 647-conjugated secondary antibody (1:500, Jackson ImmunoResearch Laboratories, 111–606-045) at room temperature for 1 h, followed by DAPI (1 μg/ml) staining for 15 min. The fluorescent signals of mCherry::H2B (in OD426) and GFP::HIS-72 (in FAS46) were detectable after methanol fixation and were measured without antibody incubation. Mounting was done using ProLong gold antifade reagent (Life Technologies). Images were acquired from Zeiss LSM 780 upright confocal microscope with a Plan-Apochromat 40 × 1.4 Oil DIC M27 objective and PMT detectors or Zeiss LSM800 with 40 × 1.4 oil DIC, 2 single PTMs and Airyscan (32-channel GaAsp PMTs). Embryos were captured as z stacks with a z-step size at 0.4 μm and 3.15 μs of pixel dwell time. Stacks with 30–35 × 0.4 μm planes were scanned for each embryo in a 3x zoom. DAPI, FITC, Cy3 and Alexa647 channels were scanned with 32 μm pinhole, and the images were saved in 16 bits format.

### 5-Ethylnyl-2′-deoxyuridine (EdU) staining of one-cell stage embryos

To permeabilize embryos, L4 worms were grown on *perm-1* dsRNA-expressing bacteria diluted 1/6 with OP50 for 24 h to achieve partial depletion (14). EdU staining of embryos for 15 min was performed as described previously (15).

### Image signal quantification

Images were processed with Fiji 2.0.0. For immunofluorescence, 31 z-sections were acquired with a spacing of 0.4 μm for each embryo. The region of interest (ROI) and the number of z-stacks for each target object were manually selected. A larger area enclosing the whole ROI within the embryo was drawn in each sample (ROI-L). Integrated density (IntDen) equals to area times mean grey value. For each channel, the integrated density of the ROI and ROI-L from all selected z-stacks containing the target object were summed (ROI^IntDen^ and ROI-L^IntDen^). The area between ROI and ROI-L were used for calculating the mean grey value of background following the equation Bg^mean^ = (ROI-L^IntDen^ - ROI^IntDen^) / (ROI-L^area^ - ROI^area^). The corrected integrated density of each targeted protein, histone modification or EdU was calculated by ROI^Corr.IntDen^ = ROI^IntDen^ – (ROI^area^ x Bg^mean^). ROI^Corr.IntDen^ was then normalized with the corrected integrated density of DAPI. The ROI^Corr.IntDen^ of ACs were normalized to the ROI^Corr.IntDen^ of endogenous chromosomes within the same embryo for comparing the signal of targeted proteins, histone modifications or EdU on AC with that on endogenous chromosomes.

## RESULTS

### Formation of artificial chromosomes (ACs) through chromatinization and *de novo* centromerization of foreign DNA in *C. elegans* one-cell embryos

In our previous study, we observed that foreign circular, supercoiled plasmid DNA injected into *C. elegans* gonad forms ACs in embryonic cells after 4–8 h of microinjection (11). An increasing proportion of ACs acquire segregation competency after they go through several cell divisions (11). We have further tested AC formation by injecting different DNA forms, including linearized plasmid DNA, and linearized plasmid DNA mixed with sheared salmon or enzyme-digested yeast genomic DNA (16). Based on the foci size of GFP::LacI that binds to the injected LacO arrays, larger ACs are formed by concatemerization of linear DNA, as compared to those formed from circular supercoiled DNA (Supplementary Figure S1A and B). This might suggest that it is more efficient to fuse linear foreign DNA fragments than to fuse circular DNA. ACs generated by injecting linear DNA (L64xLacO; (2794 bp) acquires segregation ability faster than those generated by injecting circular (supercoiled) DNA (Supplementary Figure S1C), possibly due to the larger ACs it produces (Supplementary Figure S1D). By injecting a complex DNA mixture from sheared salmon sperm DNA without the LacO repeat sequence, we confirmed that *de novo* CENP-A^HCP-3^ deposition can also occur on such ‘complex’ AC (Supplementary Figure S1E and F). However, the AC segregation rates in repetitive ACs and complex ACs have no significant difference (16).

**Figure 1.**
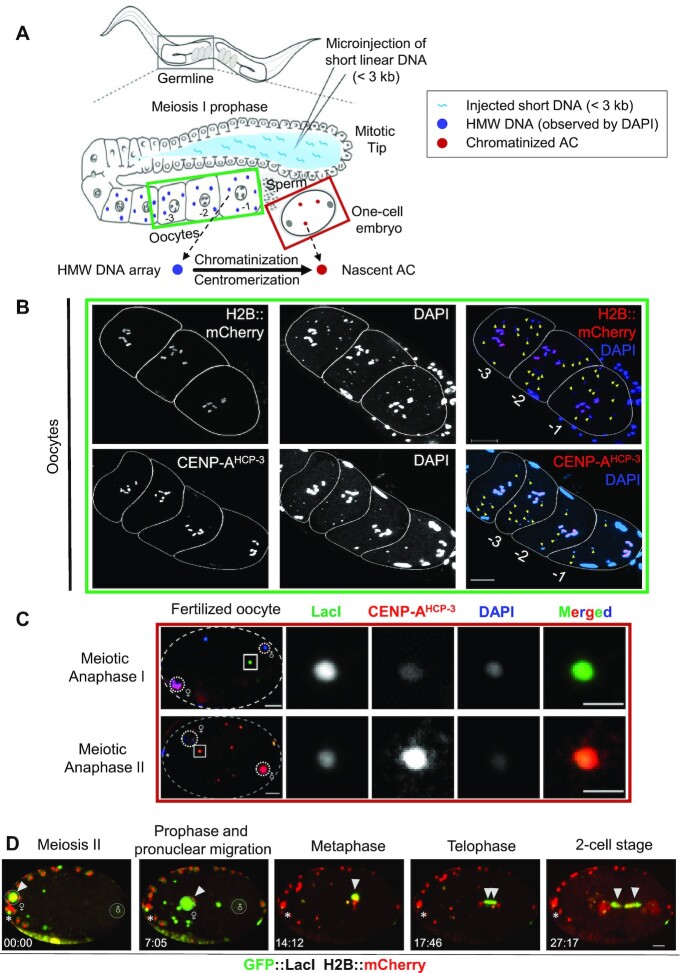
Chromatinization and *de novo* CENP-A^HCP-3^ formation in foreign HMW DNA arrays to form artificial chromosomes (ACs) in fertilized one-cell embryos. (**A**) A schematic diagram showing the delivery of short, linearized p64xLacO plasmid (L64xLacO) DNA into *C. elegans* gonad by microinjection. The foreign DNA are concatemerized to form HMW DNA arrays, which are then further chromatinized and centromerized to form artificial chromosomes (ACs). (**B**) In oocytes, DAPI stained six condensed bivalent endogenous chromosomes and multiple HMW DNA arrays, appearing as DAPI foci. Representative fluorescence images of the H2B::mCherry and immunofluorescence images of CENP-A^HCP-3^ on bivalent chromosomes. Yellow arrowheads indicate the HMW foreign DNA arrays without histone staining. Scale bar represents 10 μm. (**C**) Representative immunofluorescence images show that nascent artificial chromosomes (ACs) assembled from the HMW DNA arrays contain detectable CENP-A^HCP-3^ signals in one-cell embryos at meiosis I and II, respectively. White dash circles show the paternal and maternal DNA, and * represents the polar body. Scale bar represents 5 μm. A higher-magnification view of the representative AC (white square) is shown on the right, in which the scale bar represents 2 μm. (**D**) Time-lapse images following an AC (white arrowhead), which segregated during the first mitosis, but lagged, in one-cell embryos. The time lapses are shown (mm:ss). Scale bar represents 5 μm.

To follow the fate of foreign DNA injected into the syncytial gonad of *C. elegans*, the germline, oocytes and embryos were imaged 5 h after injection of linearized 64 copies of LacO arrays (L64xLacO). To identify the timing and location of high molecular weight (HMW) DNA array formation, DAPI (4′,6-diamidino-2-phenylindole) staining was used to stain DNA, including the concatemerized foreign DNA. Yet, in the syncytial gonad where L64xLacO was injected, no DAPI staining can be observed, suggesting that the DNA has not been concatemerized and cannot be visualized. However, punctate DAPI foci were found in the cytoplasm of the diplotene and diakinesis oocytes, suggesting that the injected DNA has fused into high molecular weight (HMW), extra-chromosomal DNA arrays (Figure 1A and Supplementary Figure S1G). Based on their smaller size, unpaired morphology and cytoplasmic location, the HMW DNA arrays can be easily distinguished from the six highly compacted endogenous bivalent chromosomes.

To determine the timing of canonical and centromeric nucleosome assembly on these DNA arrays, histone H2B and CENP-A^HCP-3^, respectively, were traced by the direct detection of the mCherry::H2B signal or the immunofluorescence signal of CENP-A^HCP-3^. These DNA arrays in oocytes lack histone H2B, CENP-A^HCP-3^ and Mis18BP1^KNL-2^ in oocytes (Figure 1B and Supplementary Figure S1G). However, in fertilized zygotes, these DNA arrays became artificial chromosomes (ACs) that contain detectable histone H2B (11) and CENP-A^HCP-3^ (Figure 1C), indicating that chromatinization and *de novo* centromerization of foreign DNA have begun in one-cell embryos after fertilization. CENP-A^HCP-3^ signal was observed on ACs as early as in embryos undergoing meiosis I (Figure 1C). Live-cell imaging showed a newly formed AC aligned at the metaphase plate and attempted to segregate during the first mitosis (Figure 1D, Supplementary Figure S1H and Supplementary Video S1). The aligned AC was pulled toward opposite poles at anaphase, but formed an anaphase bridge (Figure 1D and Supplementary Figure S2G). This result suggests that the kinetochores on newly formed ACs were sufficient to attach to the mitotic spindles to move the ACs, but error of attachment, like merotelic attachment, could cause chromosome bridge.

### Impaired DNA replication causes centromere disorganization on metaphase ACs, but does not affect *de novo* CENP-A^HCP-3^ deposition

**Figure 2.**
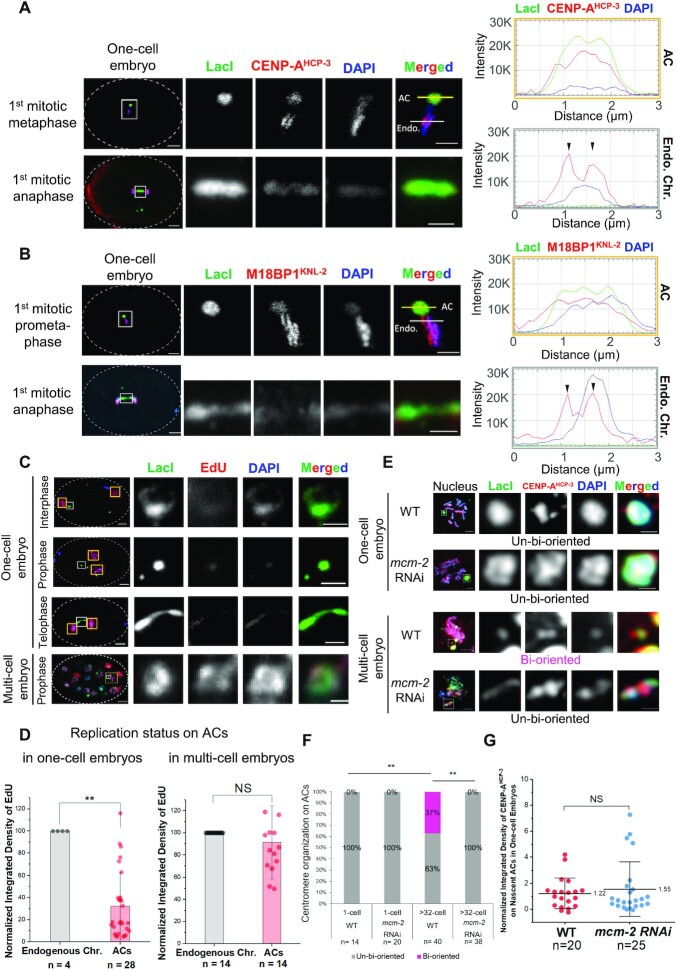
Impaired DNA replication causes centromere disorganization on ACs and anaphase bridges. (**A** and**B**) Immunofluorescence staining of ACs (LacI), inner kinetochore proteins, (A) CENP-A^HCP-3^ or (B) M18BP1^KNL-2^, and chromatin (DAPI) at metaphase and anaphase in one-cell embryos. Scale bar represents 5 μm. The selected white region is magnified on the right. A 3-μm line is drawn across the metaphase plate in the high magnification panels, and the signal intensities of CENP-A^HCP-3^ and M18BP1^KNL-2^ were measured across the AC (yellow line) and the endogenous chromosomes (white line). Scale bar in magnified panels represents 2 μm. The plot shows the line-scan signal intensities from each channel along the line. Green line: LacI; Red line: (A) CENP-A^HCP-3^ or (B) M18BP1^KNL-2^; Blue line: DAPI. The black arrowheads indicate the poleward bi-orientation of CENP-A^HCP-3^ on endogenous chromosomes. CENP-A^HCP-3^ on the AC lacks such bi-orientation at metaphase. (**C**) EdU staining of nascent ACs (LacI) in one-cell embryos at interphase, prophase and telophase, respectively, and in a multi-cell embryo. (**D**) Comparison of the average uptake of EdU after 15 min of incubation on endogenous chromosomes and nascent ACs in mitotic one-cell and multi-cell embryos, respectively. *n* equals the number of ACs or endogenous chromosomes together in one-cell or multi-cell embryos for calculating the mean of EdU integrated density. The bar chart shows the mean EdU signal (normalized to DAPI) on ACs relative to that on endogenous chromosomes. The error bars represent standard deviation (SD). Significant differences are analyzed by the Student’s *t*-test (**, *P* < 0.01; NS, not significant). (**E**) Immunofluorescence staining of CENP-A^HCP-3^ on ACs in untreated wild-type (WT) or *mcm-2* RNAi-treated one-cell and multi-cell stage embryos during prometaphase. Scale bar represents 2 μm. CENP-A^HCP-3^ distributing on the entire AC is described as ‘un-bi-oriented’, while CENP-A^HCP-3^ on the poleward sides of the AC is described as ‘bi-oriented’. (**F**) Quantification of the percentage of ACs with un-bi-oriented or bi-oriented CENP-A^HCP-3^ in one-cell and multi-cell stage WT or *mcm-2* RNAi-treated embryos. The number of ACs (*n*) analyzed was indicated. Significant differences are analyzed by the Fisher’s exact test (**, *P* < 0.01). (**G**) A scatter plot shows the quantification of integrated density of CENP-A^HCP-3^ signal on nascent ACs in WT and in *mcm-2* RNAi-treated one-cell embryos. The number of ACs (*n*) analyzed was indicated. The error bars represent standard deviation (SD). Significant differences are analyzed by the Student’s *t*-test (NS, not significant).

Proper sister chromatid segregation depends on the bi-orientation of kinetochores on sister chromatids, and the capture of microtubules emanating from opposite centrosomes. Un-bi-oriented kinetochore arrangement may facilitate merotelic attachments of microtubules and result in lagging chromosomes. To elucidate the cause of anaphase bridge formation of nascent ACs, we investigate their centromere protein level and orientation. Interestingly, we found that the initially formed centromeres, containing CENP-A^HCP-3^ and Mis18BP1^KNL-2^, inner centromeric protein, AIR-2, and condensin component SMC-4, were distributed throughout nascent ACs at metaphase (Figure 2A and B; Supplementary Figure S2A). SMC-4 has been shown to dissociate from the properly segregated endogenous chromatin during telophase (17). In contrast, high levels of SMC-4 still appeared in the center of the AC chromatin bridges at telophase (Supplementary Figure S2B).

Replicative stress induced by hydroxyurea (HU) causes excessive chromatin bridge formation (18). DNA replication has been shown to be needed for chromatin decondensation in *C. elegans* embryos during anaphase (15), which is coincident with the disassociation of condensin component SMC-4. We found that impairing DNA replication by HU treatment also results in the persistent presence of SMC-4 on the bridging endogenous chromosomes at telophase (Supplementary Figure S2C), which resembles the phenomenon of nascent AC segregation with bridges. We speculated that incomplete DNA replication may cause nascent ACs lagging. We evaluated the DNA replication efficiency on ACs by their 5-ethynyl-2′-deoxyuridine (EdU) incorporation efficiency in different embryonic stages. In one-cell stage embryos, ACs showed only 32% of EdU incorporation when compared to that on endogenous chromosomes (Figure 2C and D), which suggests that DNA replication is inefficient for nascent ACs in early-stage embryos. DNA replication is supposed to finish in S phase, so we anticipate that the DNA replicative helicase complex, MCM-2–7, to have dissociated from endogenous chromosomes before metaphase. However, live-cell imaging shows that MCM-4::mCherry has a prolonged association with ACs (Supplementary Figure S2D), suggesting that the process of DNA replication on nascent ACs was not yet complete even at metaphase. By injecting worms without GFP::LacI expression, lagging chromatin was still observed in the one-cell embryos produced (Supplementary Figure S2E), suggesting that GFP::LacI-tethering *per se* on ACs is not the source of the AC segregation problem. The sister chromatids and the kinetochores of nascent ACs have not been resolved to become bi-oriented, which may result in microtubule misattachments, lagging ACs and AC missegregation in early-stage embryos.

In multi-cell embryos, we found that the DNA replication efficiency of ACs has improved, since the EdU incorporation rate on ACs is comparable to that on endogenous chromosomes (Figure 2C and D). Consistently, more ACs in multi-cell stage embryos (37%) possessed bi-orientated centromeres than in one-cell stage wild-type embryos (0%) (Figure 2E and F), which is coincident with the increased number of evenly segregated ACs in multi-cell stage embryos (Supplementary Figure S2F and G).

MCM-2, a component of the MCM-2–7 replicative helicase, is essential for DNA replication (19), and it is also a histone chaperone for restoring histones to newly synthesized DNA (20). As the formation of the MCM-2–7 complex depends on each of its subunits, depleting MCM-2 prevents MCM-2–7 complex assembly and blocks the process of DNA replication (19). After *mcm-2* RNAi treatment, the efficiency of the depletion is confirmed by embryonic lethality (data not shown) and reduced level of *mcm-2* transcript by RT-qPCR (Supplementary Figure S5A). However, CENP-A^HCP-3^ level on endogenous chromosomes is similar in the untreated and *mcm-2* RNAi-treated one-cell embryos (Supplementary Figure S2H and I), consistent with previous work that CENP-A^HCP-3^ deposition is independent on DNA replication (21). MCM-2 depletion did not prevent ACs from aligning at the metaphase plate and segregating with the endogenous chromosomes (Supplementary Figure S2J and K), although some are missegregated. Quantification of the normalized intensity of CENP-A^HCP-3^ on nascent ACs shows no significant difference between the untreated and *mcm-2* RNAi-treated one-cell embryos (Figure 2E and G), suggesting that *de novo* CENP-A^HCP-3^ deposition is independent of MCM-2. Moreover, BUB-1, the outer kinetochore and spindle checkpoint component (22), was recruited to nascent ACs in the absence of DNA replication (Supplementary Figure S2L), which suggests that the assembly of kinetochore is independent of DNA replication.

### Condensin II facilitates *de novo* CENP-A^HCP-3^ deposition on nascent ACs in one-cell embryos

**Figure 3.**
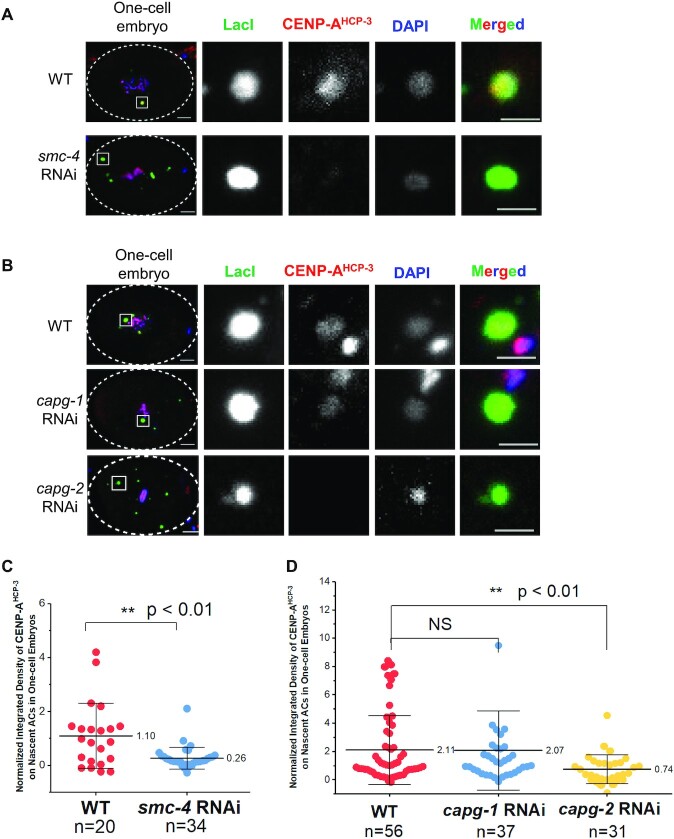
Depletion of condensin II reduces *de novo* CENP-A^HCP-3^ level on nascent ACs. (**A** and**B**) Immunofluorescence of CENP-A^HCP-3^ on ACs in WT and (A) *smc-4* RNAi-treated and (B) *cpag-1 or cpag-2* RNAi-treated one-cell embryos. Embryos were stained with antibodies against LacI (green), CENP-A^HCP-3^ (red) and DAPI (blue). Scale bar represents 5 μm. A higher magnification view of the AC (white square) is shown on the right. Scale bar represents 2 μm for the magnified images. (**C** and**D**) Scatter plots show the quantification of normalized integrated density of CENP-A^HCP-3^ signal on ACs in WT and (C) *smc-4* RNAi-treated and (D) *cpag-1 or cpag-2* RNAi-treated one-cell embryos. The integrated density was normalized with that of DAPI. The number of samples (*n*) analyzed is indicated. The error bars represent SD. Significant differences are analyzed by the Student’s *t*-test, **, *P* < 0.01.

In *C. elegans*, condensin II complex co-localizes with centromere proteins on metaphase chromosomes (17,23), and is proposed to have a specific function at the centromere in addition to chromatin condensation. However, depletion of condensin I and II component SMC-4, condensin I subunit CAPG-1 or condensin II subunit CAPG-2 has undetectable effect on CENP-A^HCP-3^ deposition for endogenous chromosomes (Supplementary Figure S3), despite that SMC-4 and CAPG-2 depletion caused lagging chromosomes and chromosome missegregation (17,23) (Supplementary Figure S3A). In human cells and *Xenopus* egg extracts, condensin II is required for new CENP-A deposition in mitotic cells and new CENP-A loading in the first mitosis, respectively (24,25). As the SMC-4 signal is positive on nascent ACs, we further determined if condensin contributes to *de novo* centromere formation on ACs in *C. elegans* embryos. We depleted CAPG-1, CAPG-2 and SMC-4, respectively. Depletion of either SMC-4 or CAPG-2 reduces the level of *de novo* CENP-A^HCP-3^ on nascent ACs (Figure 3). In contrast, depletion of CAPG-1 does not affect *de novo* CENP-A deposition on ACs (Figure 3B and D). These results suggest that condensin II may facilitate *de novo* CENP-A^HCP-3^ deposition on nascent ACs in *C. elegans* embryos.

### The spectrum of histone post-translational modifications (PTMs) on nascent ACs

**Figure 4.**
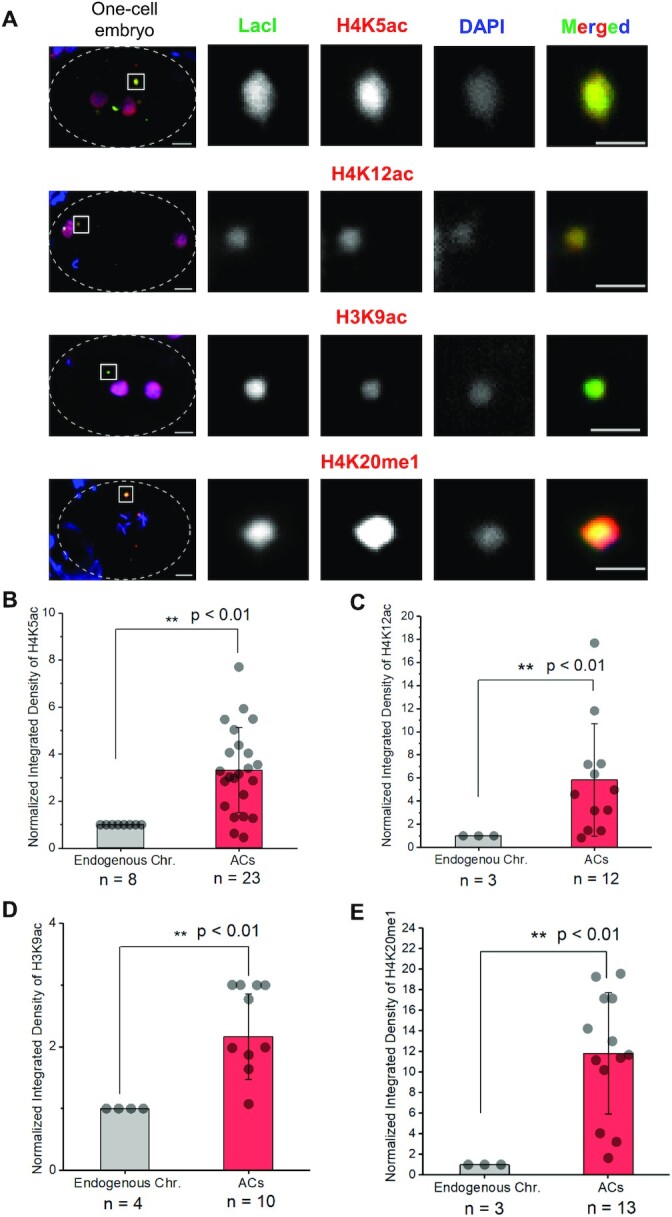
Profiling of histone post-translational modifications (PTMs) on nascent ACs in one-cell embryos by immunofluorescence staining. (**A**) Representative immunofluorescence images of H4K5ac, H4K12ac, H3K9ac and H4K20me1, on endogenous chromosomes and newly formed ACs in one-cell embryos. Embryos were stained with antibody against LacI (green), antibodies against a histone PTM (red) and DAPI (blue). Scale bar represents 5 μm. A higher-magnification view of the ACs (white square) is shown on the right. Scale bar represents 2 μm for the magnified images. The box plot shows the quantification result of the normalized integrated density of (**B**) H4K5ac, (**C**) H4K12ac, (**D**) H3K9ac or (**E**) H4K20me1 signal on endogenous chromosomes and on ACs in one-cell embryos. Only quantifications of the enriched PTMs are shown. Other PTM levels are summarized in Table 1. For quantification of PTMs on ACs and endogenous chromosomes, the signal density of each PTM was normalized with that of DAPI. The number of samples (*n*) analyzed is indicated. The error bars represent SD. Significant differences are analyzed by the Student’s *t*-test (*, *P* < 0.05; **, *P* < 0.01).

We have previously shown that H4, H3K9 acetylation and RNAPII docking facilitate *de novo* centromere formation in ACs formed by injecting circular, supercoiled p64xLacO plasmid (26). To further identify essential factors in *de novo* centromere formation, we profiled the histone PTMs on the nascent ACs generated from linear L64xLacO. We hypothesize that histone codes that co-exist with chromatinization and centromerization of newly formed ACs in one-cell embryos may lead to the identification of the required factors. We chose several histone PTMs that have been reported to be associated with centromere function. The spectrum of histone PTMs on newly formed ACs in one-cell embryos by immunofluorescence analysis (Figure 4A and Supplementary Figure S4) is summarized in Table 1. For PTMs that are associated with transcription activity, we analyzed H3K4me1 (27,28), H3K4me2 (28,29) and H3K4me3 (29,30) on newly formed ACs (Supplementary Figure S4). A medium level of H3K4me1 was present on ACs, even though H3K4me2 and H3K4me3 were lacking, suggesting that ACs might be actively transcribing. Consistently, the signal intensities of H4K5ac (31,32) (Figure 4B), H4K12ac (31,32) (Figure 4C), H3K9ac (33) (Figure 4D) and H4K20me1 (34,35) (Figure 4E) on nascent ACs were significantly higher than that on endogenous chromosomes. Meanwhile, the DNA replication-associated histone PTM, H3K56ac (20,36), on nascent ACs has dimmer signal intensity as compared to that on endogenous chromosomes (Supplementary Figure S4), consistent with the above finding that DNA replication is less efficient on nascent ACs than on endogenous chromosomes (Figure 2C and D). Moreover, heterochromatin-associated histone PTMs, including H3K9me2 (37), H3K9me3 (38) and H3K27me3 (27), were undetectable on nascent ACs in one-cell embryos (Supplementary Figure S4), consistent with our previous finding that heterochromatin is dispensable for *de novo* centromere formation (11).

**Table 1.** Summary of the profile of histone PTMs on newly formed ACs in one-cell embryos

CENP-A and histone PTMs	Signal intensity on newly formed ACs*	Function in Centromere	Associated histone modifiers or factors	Reference on function and associated factors
CENP-A^HCP-3^	++	Epigenetic mark of centromere	M18BP1^KNL-2^ and RbAp46/48^LIN-53^	(1)
H4K20me1	+++	CENP-A nucleosome	SET-1	(34,35)
H4K5ac	+++	Newly synthesized histone H4	RbAp46/48^LIN-53^ and HAT-1	(31,32)
H4K12ac	+++	Newly synthesized histone H4	RbAp46/48^LIN-53^ and HAT-1	(31,32)
H3K9ac	+++	Newly synthesized histones/ open chromatin marker	RbAp46/48^LIN-53^, HAT-1 and unknown factors	(33)
H3K56ac	+	Newly synthesized histones deposited during DNA replication	MCM-2 and ASF-1	(20,36)
H3K4me1	+	Permissive transcription	SET-17	(27,28)
H3K4me2	−	Active transcription	ASH-2, SET-17 and SET-30	(28,29)
H3K4me3	−	Robust transcription	ASH-2, SET-2 and WDR-5	(29,30)
H3K9me2	−	Heterochromatin mark	MET-2	(37)
H3K9me3	−	Heterochromatin mark	SET-25	(38)
H3K27me3	−	Heterochromatin mark	MES-2	(27)

+++: signal on newly formed ACs is significantly higher than that on endogenous chromosomes.

++: signal on newly formed ACs is comparable to that on endogenous chromosomes.

+: signal on newly formed ACs is significantly lower than that on endogenous chromosomes.

−: signal on newly formed ACs is undetectable.

### RbAp46/48^LIN-53^ is essential for chromatinization of nascent ACs

**Figure 5.**
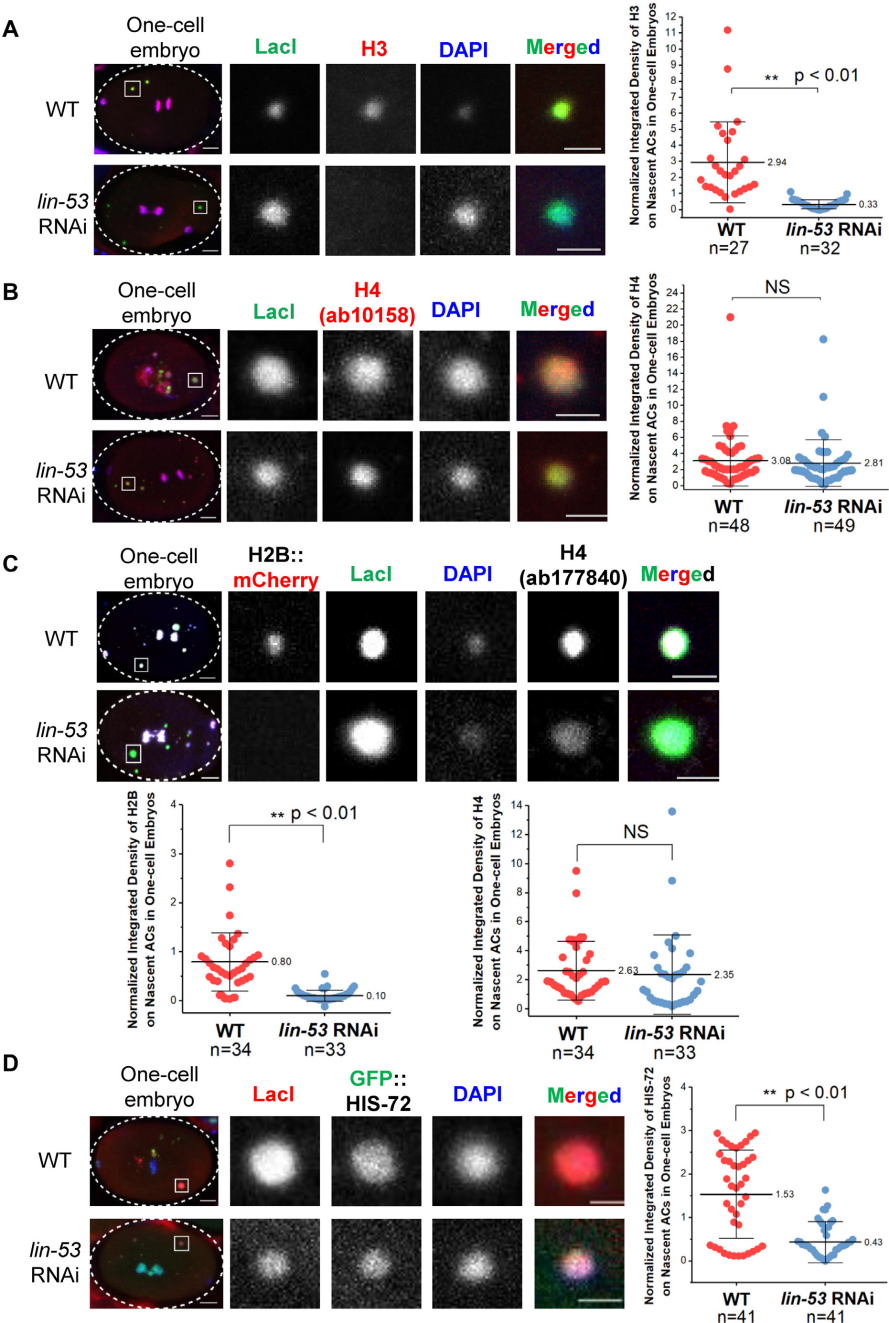
RbAp46/48^LIN-53^ is essential for chromatinization of nascent ACs. Representative images of immunofluorescence of (**A**) histone H3 and (**B**) histone H4 (ab10158), (**C**) mCherry::H2B and H4 (ab177840) and (**D**) GFP::HIS-72 (H3.3) on nascent ACs in WT and *lin-53* RNAi-treated one-cell embryos. Embryos were stained with antibody against LacI (green), antibodies against histone H4 (ab10158: red; ab177840: white) and DAPI (blue). A higher-magnification view of the AC (white square) is shown on the right. Scale bar represents 2 μm for the magnified images. Scatter plots show the quantification of normalized integrated density of (A) H3, (B) H4 (ab10158) (C) mCherry::H2B and H4 (ab177840) and (D) GFP::HIS-72 on nascent ACs. The integrated density of each histone was normalized to that of DAPI. The number of samples (*n*) analyzed is indicated. The error bars represent SD. Significant differences are analyzed by Student’s *t*-test (**, *P* < 0.01; NS, not significant).

On endogenous chromosomes, RbAp46/48^LIN-53^ also affects H2B level, though not as severe as the effect on CENP-A^HCP-3^ level (39). As RbAp46/48^LIN-53^ is also known to be a H3-H4 chaperone (40–42), and, we further tested if RbAp46/48^LIN-53^ specifically deposits acetylated histone or affects general histone deposition on nascent ACs. We found that RbAp46/48^LIN-53^ is essential for total histone H3 and H2B deposition on nascent ACs, but surprisingly not histone H4 (Figure 5A–C). The immunofluorescence experiments for H4 were repeated with two different anti-histone H4 antibodies (ab10158 and ab177840) (Figure 5B and C).

We speculated that in the absence of RbAp46/48^LIN-53^, H3 might be replaced by another major H3 variant, H3.3, which depends on HIRA-1 for deposition in *C. elegans* (43). To test if depletion of RbAp46/48^LIN-53^ affects H3.3 level of ACs, we injected L64xLacO into a strain expressing GFP::HIS-72 (H3.3). Surprisingly, we found that RbAp46/48^LIN-53^ depletion also reduced the level of GFP::HIS-72 on nascent ACs to 30% (Figure 5D), as compared with 11% of H3. It is possible that in the absence of RbAp46/48^LIN-53^, remaining H3 or H3.3, and other H3 variants could form tetramers with H4 on the injected foreign DNA, and does not affect the total level of H4 on nascent ACs. These results indicate that RbAp46/48^LIN-53^ is essential for chromatinization of foreign DNA.

### The AC segregation and the enrichment of H4K5ac, H4K12ac and H3K9ac on ACs depend on RbAp46/48^LIN-53^ and HAT-1

**Figure 6.**
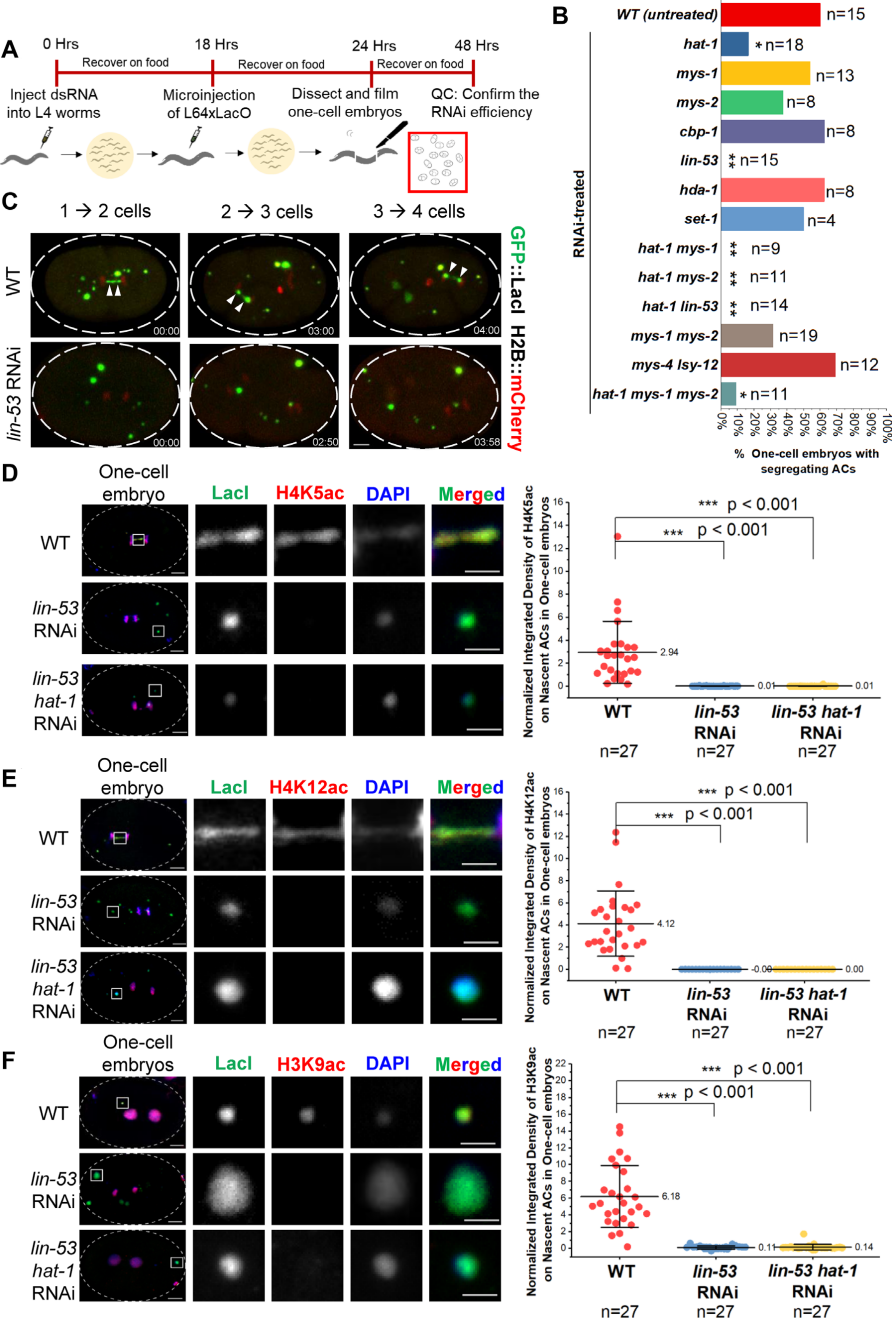
The segregation ability of nascent ACs and the enrichment of H4K5ac, H4K12ac and H3K9ac on ACs depend on RbAp46/48^LIN-53^ and HAT-1. (**A**) A schematic diagram of the experimental approach used to identify factors responsible for nascent AC segregation by RNAi and live-cell imaging. (**B**) Quantification of AC segregation rates in WT (untreated), *hat-1, mys-1, mys-2, cbp-1, lin-53, hda-1, set-1, hat-1 mys-1* double*, hat-1 mys-2* double*, mys-1 mys-2* double*, hat-1 lin-53* double, *mys-4 lsy-12* double *and hat-1 mys-1 mys-2* triple RNAi-treated one-cell embryos. Significant differences are analyzed by the Fisher’s exact test (*, *P* < 0.05; **, *P* < 0.01). The number of samples (*n*) analyzed was indicated. (**C**) Representative live-cell imaging of a nascent AC that was attempting to segregate (even with anaphase bridges) in WT (untreated) and in *lin-53* RNAi-treated one-cell embryos. The time-lapses are shown (mm:ss). Scale bar represents 5 μm. Immunofluorescence of (**D**) H4K5ac, (**E**) H4K12ac and (**F**) H3K9ac on nascent ACs in WT, *lin-53* RNAi-treated and *lin-53 hat-1* double RNAi-treated one-cell embryos. Embryos were stained with antibody against LacI (green), antibodies against a histone PTM (red) and DAPI (blue). A higher-magnification view of the AC (white square) is shown on the right. Scale bar represents 2 μm for the magnified images. Scatter plots show the quantification of normalized integrated density of (D) H4K5ac, (E) H4K12ac and (F) H3K9ac on ACs. The integrated density of each PTM was normalized to DAPI. The number of samples (*n*) analyzed is indicated. The error bars represent SD. Significant differences are analyzed by Student’s *t*-test (***, *P* < 0.001).

To investigate whether the corresponding histone modifying enzymes of the enriched AC PTMs facilitate *de novo* centromere formation, we performed RNA interference (RNAi) by injecting dsRNA of candidate histone modifier genes to L4 stage worms expressing GFP::LacI and mCherry::H2B (Figure 6A). The RNAi efficiency of each gene was confirmed by RT-qPCR (Supplementary Figure S5A) or live-cell imaging (Supplementary Figure S5B). Eighteen hours after dsRNA injection, L64xLacO was injected to RNAi-treated worms or untreated worms of the same stage. Embryos were dissected from injected worms and mounted for live-cell imaging to measure the AC segregation rate, and for immunofluorescence analysis to compare the CENP-A^HCP-3^ signal on ACs.

We depleted individual histone acetyltransferases (*hat-1, cbp-1, mys-1, mys-2, lsy-12* and *mys-4*), a histone deacetylase (*hda-1*), a histone methyltransferase (*set-1*) that is responsible for H4K20me1 (44), or depleted them in double and triple combinations. For untreated controls, the percentage of one-cell embryos that have segregating ACs among all one-cell embryos with ACs is 60%, including segregating ACs with anaphase bridges. Among all single RNAi treatments of acetyltransferases, *hat-1* RNAi significantly reduces AC segregation frequency to 17% (*P* < 0.05) (Figure 6B). Single RNAi of other acetyltransferases (*cbp-1, mys-1, mys-2*) does not cause any significant decrease in the AC segregation rate. Double knockdown of *hat-1 mys-1* and *hat-1 mys-2*, and triple knockdown of *hat-1 mys-1 mys-2* further decrease AC segregation rates in one-cell stage embryos (Figure 6B). These findings indicate that acetyltransferases play an essential role in the segregation of nascent ACs. MYS-1 and MYS-2 may share some overlapping acetylation targets with HAT-1, and thus have additive effects upon depletion. In contrast, the AC segregation rates in *mys-1 mys-2* knockdown and *lsy-12 mys-4* knockdown embryos have no significant difference with WT embryos (Figure 6B). Depletion of HDA-1 or SET-1 also did not affect AC segregation in one-cell embryos (Figure 6B).

Interestingly, depletion of RbAp46/48^LIN-53^, the CENP-A^HCP-3^ chaperone (39) and the HAT-1 physical interactor (Supplementary Figure S5D), completely abolished the segregation competency of nascent ACs (Figure 6B and C). Live-cell imaging videos (Supplementary Videos S3 and S4) show examples of an untreated and a *lin-53* RNAi-treated embryo, both containing ACs, and they went through the first three consecutive cell divisions from one-cell stage to four-cell stage. A nascent AC in a WT embryo aligned at the metaphase plate with endogenous chromosomes, attempted to segregate, but formed chromosome bridges at the first anaphase in one-to-two cell stage, and has less severe chromosome lagging during the three-to-four cell transition. In contrast, all nascent ACs were passively remained in one of the two daughter cells during each division in *lin-53* RNAi-treated embryo. Either RbAp46/48^LIN-53^ or HAT-1 depletion, or double depletion significantly reduces the level of H4K5ac, H4K12ac and H3K9ac on ACs (Figure 6D–F and Supplementary Figure S5E-G). The results suggest that RbAp46/48^LIN-53^ and HAT-1 may facilitate histone H4 acetylation at these sites or preferentially deposit acetylated histones. On the other hand, as RbAp46/48^LIN-53^ affects total H3 level on ACs in addition to H3K9ac, it is difficult to conclude which step functions in.

### HAT-1 assists RbAp46/48^LIN-53^ in *de novo* CENP-A^HCP-3^ deposition on nascent ACs

**Figure 7.**
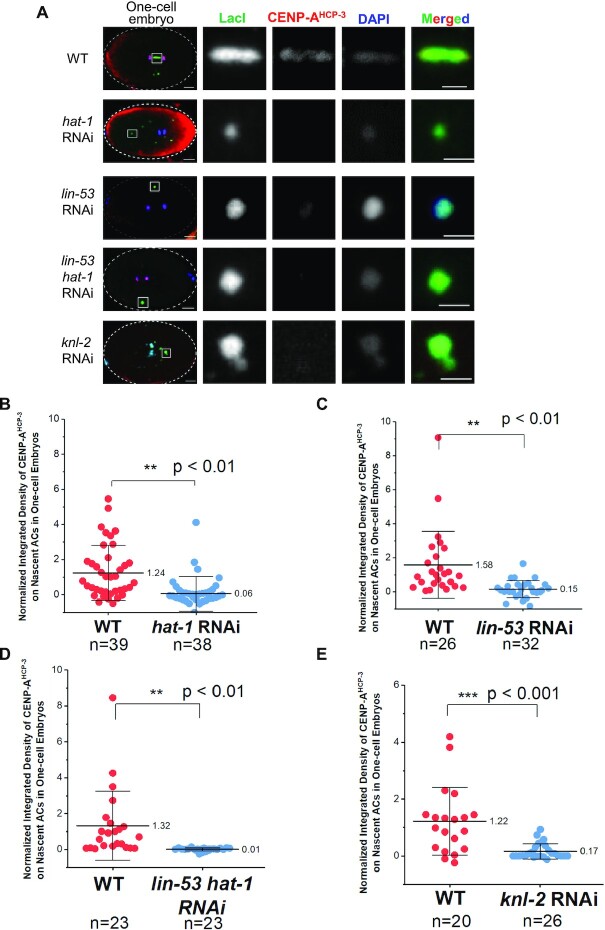
HAT-1 assists RbAp46/48^LIN-53^ in *de novo* CENP-A^HCP-3^ deposition on nascent ACs. (**A**) Immunofluorescence of CENP-A^HCP-3^ on ACs in WT, *hat-1* RNAi, *lin-53*, *lin-53 hat-1* double and *knl-2* RNAi-treated one-cell embryos. A higher-magnification view of the ACs (white square) is shown on the right. Scale bars in whole embryo images and in the magnified images represent 5 and 2 μm, respectively. (**B–E**) Scatter plots show the quantification of the normalized integrated density of CENP-A^HCP-3^ signal on ACs in (B) *hat-1*, (C) *lin-53*, (D) *lin-53 hat-1* double and (E) *knl-2* RNAi-treated one-cell embryos, compared with that in WT embryos. The integrated density of CENP-A^HCP-3^ was normalized to DAPI. The number of samples (*n*) analyzed is indicated. The error bars represent SD. Significant differences are analyzed by the Student’s *t*-test (**, *P* < 0.01).

RbAp46/48^LIN-53^ deposits CENP-A^HCP-3^ on endogenous chromosomes, but HAT-1 depletion has no effect on CENP-A^HCP-3^ on endogenous chromosomes (39). Since depletion of HAT-1, RbAp46/48^LIN-53^ and double depletion of RbAp46/48^LIN-53^ and HAT-1 significantly decreased AC segregation rate, we proposed that RbAp46/48^LIN-53^ and HAT-1 are responsible for depositing CENP-A^HCP-3^-H4 tetramers on nascent ACs after fertilization. We performed immunofluorescence analysis of CENP-A^HCP-3^ on the nascent ACs in untreated, *hat-1, lin-53* and *lin-53 hat-1* double RNAi-treated embryos, and found that the CENP-A^HCP-3^ level on ACs is significantly decreased in *hat-1* and *lin-53* and double RNAi-treated embryos (Figure 7A–D).

### RbAp46/48^LIN-53^ is required for *de novo* Mis18BP1^KNL-2^ localization on nascent ACs

**Figure 8.**
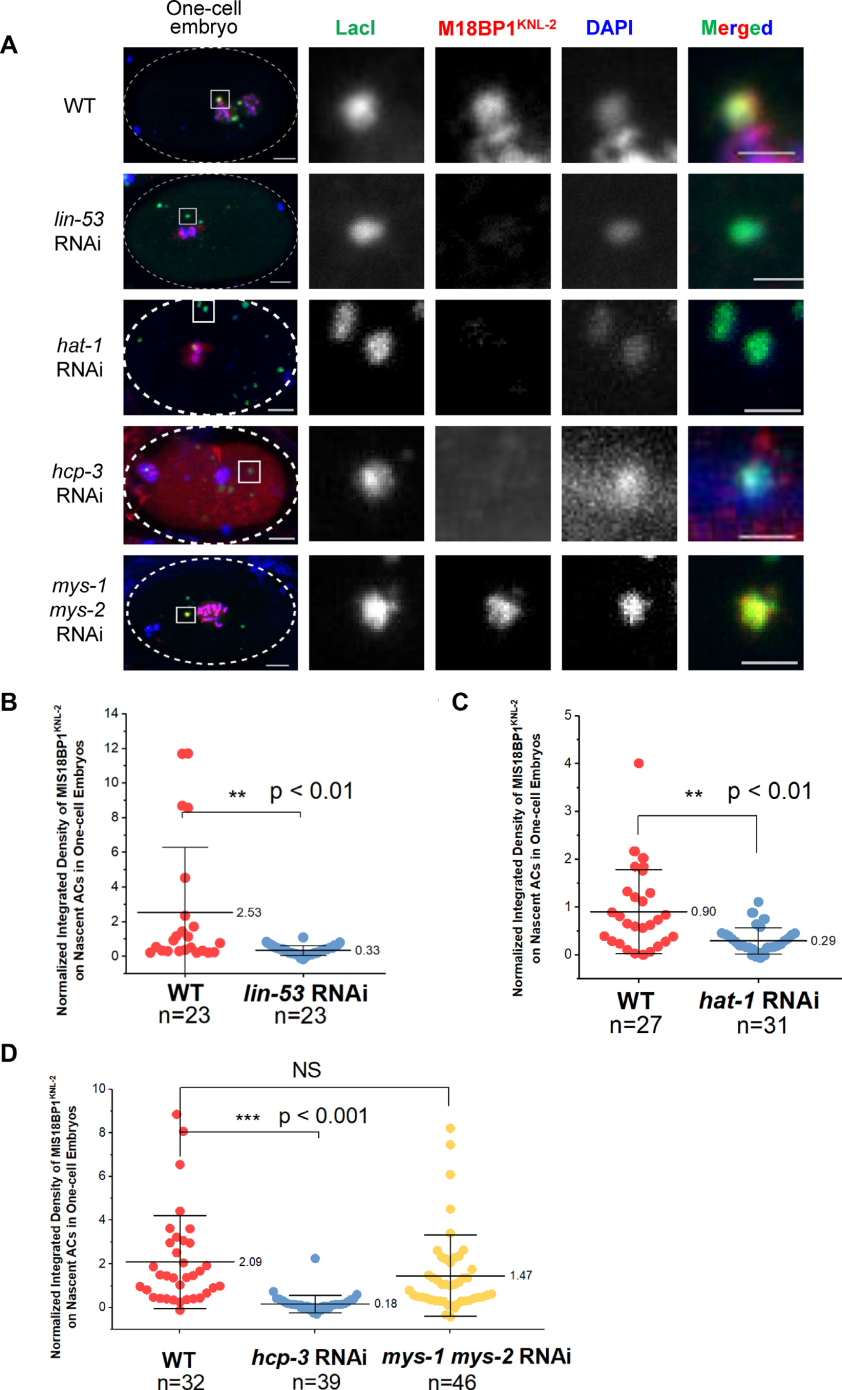
RbAp46/48^LIN-53^-initiated *de novo* CENP-A^HCP-3^ deposition is required for Mis18BP1^KNL-2^ localization. (**A**) Immunofluorescence of M18BP1^KNL-2^ on ACs in WT, *lin-53*, *hcp-3* and *mys-1 mys-2* double RNAi-treated one-cell embryos. A higher-magnification view of the ACs (white square) is shown on the right. Scale bars in whole embryo images and in the magnified images represent 5 and 2 μm, respectively. (**B–D**) Scatter plots show the quantification result of the normalized integrated density of M18BP1^KNL-2^ signal on ACs in (B) *lin-53*, (C) *hat-1*, and (D) *hcp-3* and *mys-1 mys-2* double RNAi-treated one-cell embryos, compared with that in WT embryos. The integrated density of M18BP1^KNL-2^ was normalized to that of DAPI. The number of samples (*n*) analyzed is indicated. The error bars represent SD. Significant differences are analyzed by the Student’s *t*-test (**, *P* < 0.01; ***, *P* < 0.001).

Mis18BP1^KNL-2^ and CENP-A^HCP-3^ are interdependent for each other's localization on endogenous chromosomes of *C. elegans* (45). To test if Mis18BP1^KNL-2^ is also necessary for *de novo* CENP-A^HCP-3^ deposition on nascent ACs, we depleted Mis18BP1^KNL-2^ and performed immunofluorescence analysis. *knl-2* RNAi almost completely abolished CENP-A^HCP-3^ signal on nascent ACs (Figure 7A and E). This indicates that Mis18BP1^KNL-2^ is also essential for CENP-A^HCP-3^ localization on both nascent ACs and endogenous centromeres. Similar to endogenous centromeres, Mis18BP1^KNL-2^ localization on nascent ACs also relies on CENP-A^HCP-3^ (Figure 8A and D). We simultaneously stained CENP-A^HCP-3^ and Mis18BP1^KNL-2^ on nascent ACs in one-cell embryos, and show that 62% of ACs have both CENP-A^HCP-3^ and Mis18BP1^KNL-2^, while 38% of ACs have neither of the signals (Supplementary Figure S6A and B). We have not found any ACs that have only CENP-A^HCP-3^ or only Mis18BP1^KNL-2^, which is consistent with the co-dependence of Mis18BP1^KNL-2^ and CENP-A^HCP-3^ localization (Figures 7A, E, 8A and D).

**Figure 9.**
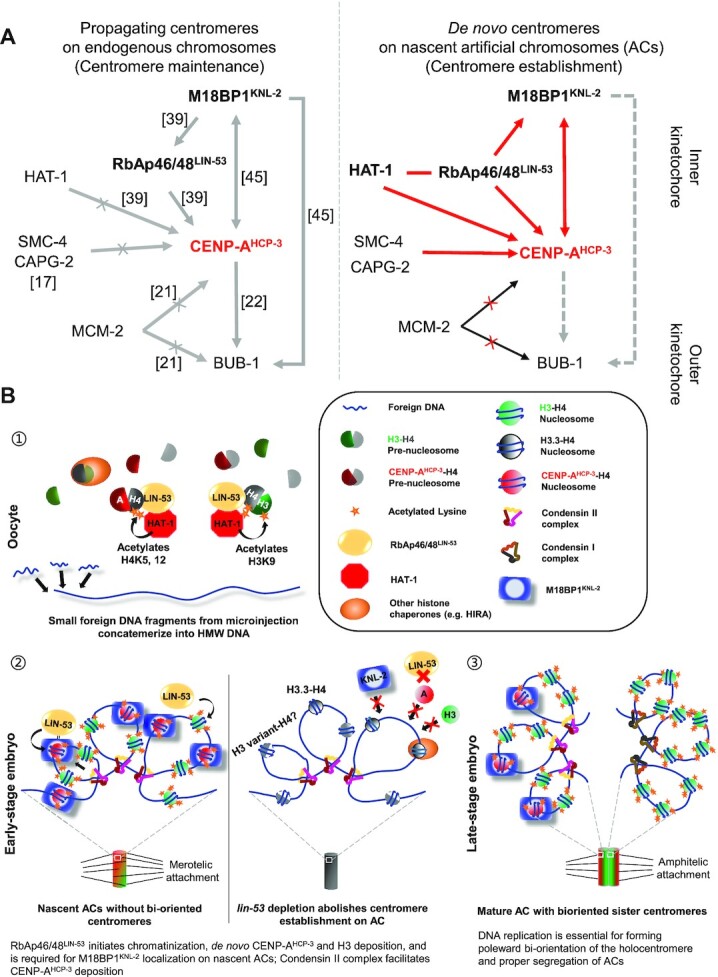
A model of *de novo* centromere formation in *C. elegans* embryos. (**A**) A schematic diagram of the centromeric protein localization dependency during centromere maintenance on endogenous chromosomes and in *de novo* centromere formation on nascent ACs in *C. elegans*. A→B means B’s localization is dependent on A. Gray arrows indicate the findings from other studies. Red arrows indicate the findings from this study. Dashed arrows indicate that the dependency is predicted, but not confirmed. The line between two factors indicates that they have physical interaction. (**B**) The proposed process of artificial chromosome formation in *C. elegans* gonad. 1. First, small foreign DNA fragments from microinjection concatemerize into HMW DNA arrays in the oocytes. RbAp46/48^LIN-53^-HAT-1 complex acetylates H3-H4 and CENP-A-H4 pre-nucleosomes at H4K5, H4K12 and H3K9ac, which contribute to the hyperacetylation of nascent ACs. 2. Second, RbAp46/48^LIN-53^ initiates chromatinization, *de novo* CENP-A^HCP-3^ and H3 deposition, and RbAp46/48^LIN-53^ is required for M18BP1^KNL-2^ localization on HMW DNA; Condensin II complex also facilitates CENP-A^HCP-3^ deposition. Chromatinization and centromerization of the HMW DNA generates nascent ACs. Nascent ACs have DNA replication defects and lack bi-oriented sister kinetochores, which could lead to merotelic attachments to the mitotic spindle and chromosome bridging (in early embryonic cells). 3. Finally, DNA replication efficiency gradually improves on ACs, and ACs ‘mature’ in late embryonic stage. In matured ACs, bi-oriented sister kinetochores allow amphitelic attachment of spindles and proper segregation.

On endogenous chromosomes, RbAp46/48^lin-53^ depletion only reduced CENP-A^HCP-3^ level but did not affect Mis18BP1^KNL-2^ level (39). We monitored the M18BP1^KNL-2^ signal on nascent ACs in *lin-53* RNAi-treated embryos. Surprisingly, at *de novo* centromeres on nascent ACs, RbAp46/48^LIN-53^ depletion also leads to the loss of initial Mis18BP1^KNL-2^ deposition (Figure 8A and B), which suggests that while Mis18BP1^KNL-2^ could be a self-directing factor for centromere maintenance in the existing centromeres, it is downstream of RbAp46/48^LIN-53^ in *de novo* centromere establishment (Figure 9A).

Interestingly, we found that *hat-1* RNAi treatment reduces M18BP1^KNL-2^ level on nascent ACs to 30% (Figure 8A and C). In human cells, MYST2 has been described as an interactor of Mis18 complex for regulating CENP-A deposition (46). However, the function of MYS family of acetyltransferases on centromere chromatin has not been reported previously in *C. elegans*. Since we found that MYS-1 and MYS-2 depletion have additive effect with *hat-1* RNAi in abolishing AC segregation, we tested whether double knockdown of *mys-1* and *mys-2* prevents Mis18BP1^KNL-2^ localization on nascent ACs. However, the Mis18BP1^KNL-2^ signal on nascent ACs in *mys-1* and *mys-2* double depleted embryos shows no significant difference as compared with untreated embryos (Figure 8A and D).

## DISCUSSION

Chromatinized ACs were formed in fertilized embryos a few hours after microinjection of foreign circular DNA (11) and linear DNA. Histone H2B and centromeric protein CENP-A^HCP-3^ signals on ACs were detectable in fertilized oocytes and one-cell embryos (Figure 1B and C). This is consistent with the finding that major sperm proteins trigger nuclear membrane breakdown (47) and release the nuclear-localized histones and centromeric proteins to allow chromatinization and centromerization of the HMW DNA arrays, which are initially located in the cytoplasm. The whole process of AC formation is summarized in Figure 9B.

Interestingly, in wild-type, the average level of CENP-A^HCP-3^ (normalized to the amount of DNA based on DAPI staining) on nascent ACs formed from linear plasmid DNA is 1.3-fold higher than that on endogenous chromosomes in one-cell embryos (Supplementary Figure S1I). This observation is slightly different from previous study in which the level of CENP-A^HCP-3^ on ACs in one-cell stage is lower than that on endogenous chromosomes (26). However, the CENP-A^HCP-3^ signal on ACs formed from circular DNA increases quickly in the first few cell cycles to become comparable with that in endogenous chromosomes in 17–32 cell stage (26). Such difference in initial of CENP-A^HCP-3^ level could be due to the difference in size or structure of the ACs from different injected DNA forms (Supplementary Figure S1B–D).

We found that SMC-4 is present on chromosome bridges upon HU treatment, which is possibly due to the decondensation defects caused by replication stress (15), although the presence of SMC-4 could also be due to the enrichment of condensin I complex on chromatin bridges to prevent cleavage-furrow regression (48). We examined the DNA replication status on nascent ACs in one-cell embryos and found that it is less efficient than on endogenous chromosomes. In *C. elegans*, DNA replication origins contain H3K4me2 enrichment (49), whereas H3K4me2 is absent on nascent ACs. The underlying non-*C. elegans* sequences or chromatin environment of ACs could be the reason for the less efficient DNA replication on nascent ACs, but surprisingly DNA replication efficiency improves quickly in few cell cycles together with higher frequency of bi-oriented kinetochores (Figure 2F) and even segregation (Supplementary Figure S2F and G). Expectedly, *mcm-2* depletion perturbed kinetochore bi-orientation on both endogenous chromosomes and ACs (Figure 2E and F). During DNA replication, nucleosomes are disassembled from the parental DNA strand ahead of the DNA replication machinery (50). MCM-2 has been recently proposed to be able to chaperone H3-H4 and CENP-A-H4 dimers, for replenishing them to the sister chromatins behind the replication forks (20). However, *mcm-2* RNAi did not prevent the process of *de novo* CENP-A^HCP-3^ deposition on nascent ACs (Figure 2E and G), and did not affect their ability to recruit outer kinetochore and spindle checkpoint component BUB-1 (Supplementary Figure S2J). Although CENP-A^HCP-3^ was found to be almost completely turned over between two cell cycles (21), it is not clear when the new CENP-A^HCP-3^ was loaded on the holocentromere during the cell cycle in *C. elegans* embryos. We postulate that *de novo* centromere formation on foreign DNA *per se* is independent of DNA replication. Indeed, the CENP-A^HCP-3^ levels on nascent ACs (Figure 2E and G) and endogenous chromosomes (Supplementary Figure S2H), when normalized to the DNA amount (DAPI) or H2B, are similar in wild-type and *mcm-2* RNAi-treated embryos, which supports this hypothesis. Interestingly, this is similar to CENP-A replenishment on chromatin in human cells, which occurs before S phase and is independent of DNA replication (51). Yet, it is different from the case in budding yeast, in which CENP-A^Cse4^ also completely turns over and reloads to sister chromatids in S phase, dependent on DNA replication (52,53).

Condensin II, but not condensin I, is specifically enriched at the centromeres and has been found to promote CENP-A deposition in human cells and *Xenopus* oocyte extracts (24–25,54). In human cells, the interaction between condensin II and HJURP is needed for HJURP’s centromeric localization, and for depositing new CENP-A (24). On the other hand, depleting condensin II in *Xenopus* oocyte extracts reduces the CENP-A level at the centromere, but not the HJURP level (25). In *C. elegans*, condensin II subunits also co-localize with CENP-A^HCP-3^ starting at prometaphase in embryonic cells (23). Despite that condensin II depletion does not affect loading of CENP-A^HCP-3^ on endogenous chromosomes (Supplementary Figure S3), we found that depleting condensin I/II component SMC-4 and condensin II-specific component CAPG-2 reduces *de novo* CENP-A^HCP-3^ deposition on newly formed ACs (Figure 3). These findings indicate that condensin II facilitates *de novo* centromere formation. However, we could not detect physical interaction between condensin II subunit SMC-4 and CENP-A^HCP-3^ chaperone RbAp46/48^LIN-53^ by co-immunoprecipitation (Supplementary Figure S5D). Condensin II might modify or maintain the ideal chromatin structure for *de novo* CENP-A^HCP-3^ deposition.

In human and chicken DT-40 cells, H4K20me1 was reported as a histone PTM on CENP-A nucleosomes enriched at centromeres, which is essential for CENP-T localization (34). In *C. elegans*, H4K20 is monomethylated by methyltransferase SET-1 (44). Though H4K20me1 is enriched on nascent ACs, the AC segregation rate in *set-1* RNAi-treated embryos shows no significant difference from that in untreated embryos (Figure 6B), indicating that H4K20me1 is not essential for *de novo* CENP-A^HCP-3^ deposition.

We found that histone acetylation on H3K9, H4K5 and H4K12 was significantly enriched on nascent ACs formed from linear DNA, which is consistent with the previously documented acetylated H3K9 and panH4ac (on K5, 8, 12 or 16) on ACs from circular injected DNA (26). H3K9ac has been reported to be associated with the deposition of newly synthesized histones H3 in *Tetrahymena*, while the involved acetyltransferase is not clear (55). In human cells, H3K9ac also induces *de novo* CENP-A deposition on alphoid DNA at ectopic site and is compatible with centromere functioning (33). Our result shows that the level of H3K9ac (Figure 6F) and H3 (Figure 5A) were both significantly decreased in *lin-53* RNAi-treated embryos, suggesting that RbAp46/48^LIN-53^ is involved in both chromatinization and H3K9 acetylation. However, we cannot distinguish if RbAp46/48^LIN-53^ specifically deposits H3K9ac to the foreign DNA or its depletion inhibits the overall deposition of H3 including H3K9ac on foreign DNA. Notably, in species as divergent as humans, *Drosophila*, *Tetrahymena* and yeast, newly synthesized and newly deposited H4 are acetylated in a conserved pattern at lysines 5 and 12 before its association with DNA (55–57). Pre-nucleosomal H4 is di-acetylated at K5 and K12 by HAT-1 in human cells, DT-40 cells and yeast, through forming a complex with H3-H4 chaperone, RbAp46/48 (40,58–59). We observed a significant reduction of H4K5ac and H4K12ac (Figure 6D and E) on nascent ACs after *lin-53* RNAi treatment, while the total H4 level remains unchanged (Figure 5B and C). RbAp46/48^LIN-53^ may preferentially deposit acetylated H4K5,12ac nucleosomes on the foreign DNA.

We demonstrated that the physical interaction between RbAp46/48^LIN-53^ and HAT-1 were also conserved in *C. elegans* (Supplementary Figure S5D), as in *S. pombe*, *S. cerevisiae* and human cells (57,59–60). A previous study has shown that human HAT1-RbAp46 complex binds and acetylates H4 in H3.1-H4 complex more efficiently than that in H3.3-H4 complex (61). The depletion of *C. elegans* HAT-1 leads to a significant decrease in H4K5ac and H4K12ac levels on nascent ACs (Supplementary Figure S5E and F), suggesting that HAT-1 may acetylate H4 at K5 and K12 in the HAT-1-RbAp46/48^LIN-53^-H4-H3/CENP-A^HCP-3^ complex. In RbAp46/48^LIN-53^ depletion, the non-acetylated histone H4 might still form tetramer with histone H3.3 on foreign DNA. As histone H2B level is also reduced on ACs in *lin-53* depletion (Figure 5C), it may suggest that full nucleosome assembly level is lowered.

In *lin-53* RNAi-treated embryos, the unchanged level of H4 did not match with the reduced levels of H3 and H3.3 on nascent ACs. H3.3 could be deposited through other histone chaperones, like HIRA (Figure 9B) (43) and did not completely rely on RbAp46/48^LIN-53^ (62). The unmatched level of H4 and H3 variant is observed possibly because we only detected one of the H3.3 isoforms (HIS-72). Other H3.3 isoforms and other H3 variants, might also be able to form tetramer nucleosomes with H4 on foreign DNA. Besides, non-nucleosomal H4, possibly without an H3 partner, might bind to the partially chromatinized DNA, potentially causing more chromosomal instability.

In chicken DT-40 cells, RbAp48 is essential for new CENP-A deposition to the centromere, which cooperates with HAT-1 to acetylate pre-nucleosomal CENP-A-H4 complex at H4K5 and K12 (31). In *Drosophila*, an *in vitro* experiment shows that RbAp48 alone is sufficient to assemble CENP-A^CID^-H4 to the naked DNA (63). New evidences show that HAT-1 interacts directly with CENP-A^CID^ in *Drosophila* and depletion of HAT-1 reduces the efficiency of new CENP-A^CID^ deposition significantly (64). Nevertheless, in fission yeast, RbAp46/48^Mis16^ and HJURP^Scm3^ are present in the same complex, where RbAp46/48^Mis16^ distinguishes CENP-A^Cnp1^-H4 from H3-H4 by recognizing HJURP^Scm3^ and H4 independently (65). The temporal (RbAp46/48^Mis16^-CENP-A^Cnp1^-H4-HJURP^Scm3^) complex binds to the centromere-specific Mis18-Eic1 complex through Mis18-HJURP^Scm3^ interaction, then RbAp46/48^Mis16^ switches binding from H4 to Eic1 for CENP-A^Cnp1^ deposition (66). In HeLa cells, RNAi knockdown of RbAp46/48 reduces ectopic loading of CENP-A^S68E^ mutant on the chromosome arms, which cannot bind to HJURP, suggesting that RbAp46/48 may deposit CENP-A^S68E^-H4 to ectopic loci in the absence of HJURP (67). On the other hand, in oocyte extracts of *Xenopus*, RbAp48 depletion does not affect normal CENP-A incorporation to the centromere on the sperm chromatin but causes ectopic CENP-A deposition (25,31). We observed a significant reduction of the AC segregation rate in *hat-1* RNAi-, *lin-53* RNAi- and *lin-53 hat-1* double RNAi*-*treated embryos, indicating that RbAp46/48^LIN-53^ and HAT-1 are both involved in *de novo* centromere formation (Figure 6B). Indeed, we found that both RbAp46/48^LIN-53^ and HAT-1 are critical for *de novo* CENP-A^HCP-3^ deposition on *C. elegans* ACs (Figure 7A–D).

M18BP1^KNL-2^, conserved in *C. elegans*, is upstream of RbAp46/48^LIN-53^, and both are essential for CENP-A^HCP-3^ deposition in endogenous centromeres (39,45). In fission yeast, Mis-18, RbAp46/48^Mis16^ and HJURP^Scm3^ have been found in the same complex for depositing CENP-A^Cnp1^ to the centromere (65,68–69). In humans and vertebrates, CENP-A-HJURP relies on the MIS18 complex (including MIS-18α, MIS-18β and M18BP1) for centromeric targeting (70,71). Tethering LacI-fused M18BP1 or Mis-18β to the LacO region promotes new CENP-A deposition by recruiting HJURP to the tethered locus (70), similar to tethering HJURP to an ectopic locus (72). M18BP1^KNL-2^, as a priming factor, is anticipated to anchor to the existing centromeres for directing new CENP-A loading. Since *C. elegans* has no HJURP, Mis18α nor Mis18β identified so far, M18BP1^KNL-2^ alone may direct RbAp46/48^LIN-53^ to deposit CENP-A^HCP-3^ to existing centromeres. Here, we show that depleting M18BP1^KNL-2^ also significantly reduced CENP-A^HCP-3^ level on nascent ACs (Figure 7A and E), consistent with its effect on endogenous chromosomes. The inter-dependency between M18BP1^KNL-2^ and CENP-A is true for both endogenous chromosomes (39,45) and on nascent ACs. Surprisingly, in RbAp46/48^LIN-53^-depleted embryos, M18BP1^KNL-2^ was not able to localize to the nascent ACs without any pre-seeded CENP-A^HCP-3^ (Figure 8A and B; Supplementary Figure S6A and B). Although it might be possible that the chromatinization by RbAp46/48^LIN-53^ is important for M18BP1^KNL-2^ localization, our finding is also consistent with the hypothesis that RbAp46/48^LIN-53^ initiates CENP-A^HCP-3^ nucleosome assembly on foreign DNA, which lays the foundation for loading of other kinetochore proteins, including M18BP1^KNL-2^. Similarly, depletion of HAT-1 reduces M18BP1^KNL-2^ levels on nascent ACs (Figure 8A and C), which could be due to the decreased level of CENP-A^HCP-3^, or nucleosome, on them (Figure 7A and B). The centromeric protein localization dependency in *C. elegans* endogenous chromosomes and in *de novo* centromere formation on ACs has been summarized and compared (Figure 9A). It will be of great interest to know if RbAp46/48^LIN-53^ and M18BP1^KNL-2^ form a complex to deposit pre-nucleosomal CENP-A^HCP-3^-H4, in which RbAp46/48^LIN-53^ is released from the centromere after the CENP-A^HCP-3^ deposition, while M18BP1^KNL-2^ is retained on the chromatin for CENP-A^HCP-3^ stabilization and for recruiting outer kinetochore proteins.

The process of *de novo* centromere formation has been summarized in Figure 9B. Here, we proposed a hypothesis to explain the robust *de novo* centromere formation on foreign DNA in *C. elegans*, which may be associated with a single amino acid substitution of CENP-A during the evolution, and the promiscuous CENP-A^HCP-3^ deposition by RbAp46/48^LIN-53^. Phosphorylation of human CENP-A at Ser68 has been proposed to be important for preventing premature HJURP binding at metaphase in HeLa cells (67), but the function of CENP-A at Ser68 has been controversial (73). However, mutating the Ser68 residue to alanine, as a phospho-dead mutant, causes continuous CENP-A binding to HJURP and ectopic CENP-A deposition (67). Interestingly, this site is evolutionarily conserved in most eukaryotes except in budding yeast and *C. elegans*, where these CENP-A homologues have an alanine at this position, similar to the human phospho-dead mutant. In budding yeast and *C. elegans*, the CENP-A loading time may not be dependent on phosphorylation at this site at all. In budding yeast, CENP-A^Cse4^ propagation relies on HJURP^Scm3^ (52), and as a result, RbAp46/48^Hat2^ can be a non-essential protein in this species (74). On the other hand, *C. elegans* may have lost HJURP, but CENP-A^HCP-3^ deposition depends on RbAp46/48^LIN-53^ instead. For endogenous chromosome CENP-A^HCP-3^ propagation, the cue for new CENP-A^HCP-3^ deposition depends on pre-existing M18BP1^KNL-2^ at the centromere. For nascent ACs, RbAp46/48^LIN-53^ probably does not rely on pre-existing CENP-A^HCP-3^ to deposit CENP-A^HCP-3^ on the foreign DNA, facilitating *de novo* centromere formation.

## Supplementary Material

gkab217_Supplemental_Files
