# Supplementary material for: RbAp46/48LIN-53 and HAT-1 are required for initial CENP-AHCP-3 deposition and de novo holocentromere formation on artificial chromosomes in Caenorhabditis elegans embryos
Source: Nucleic Acids Res. 2021 Apr 19;49(16):9154–73. doi: 10.1093/nar/gkab217 (PMC8450102; doi:10.1093/nar/gkab217)
Supplement: gkab217_Supplemental_Files [file gkab217_supplemental_files.zip › LIN53AC_suppl.figures_20210306.pdf]

Figure S1

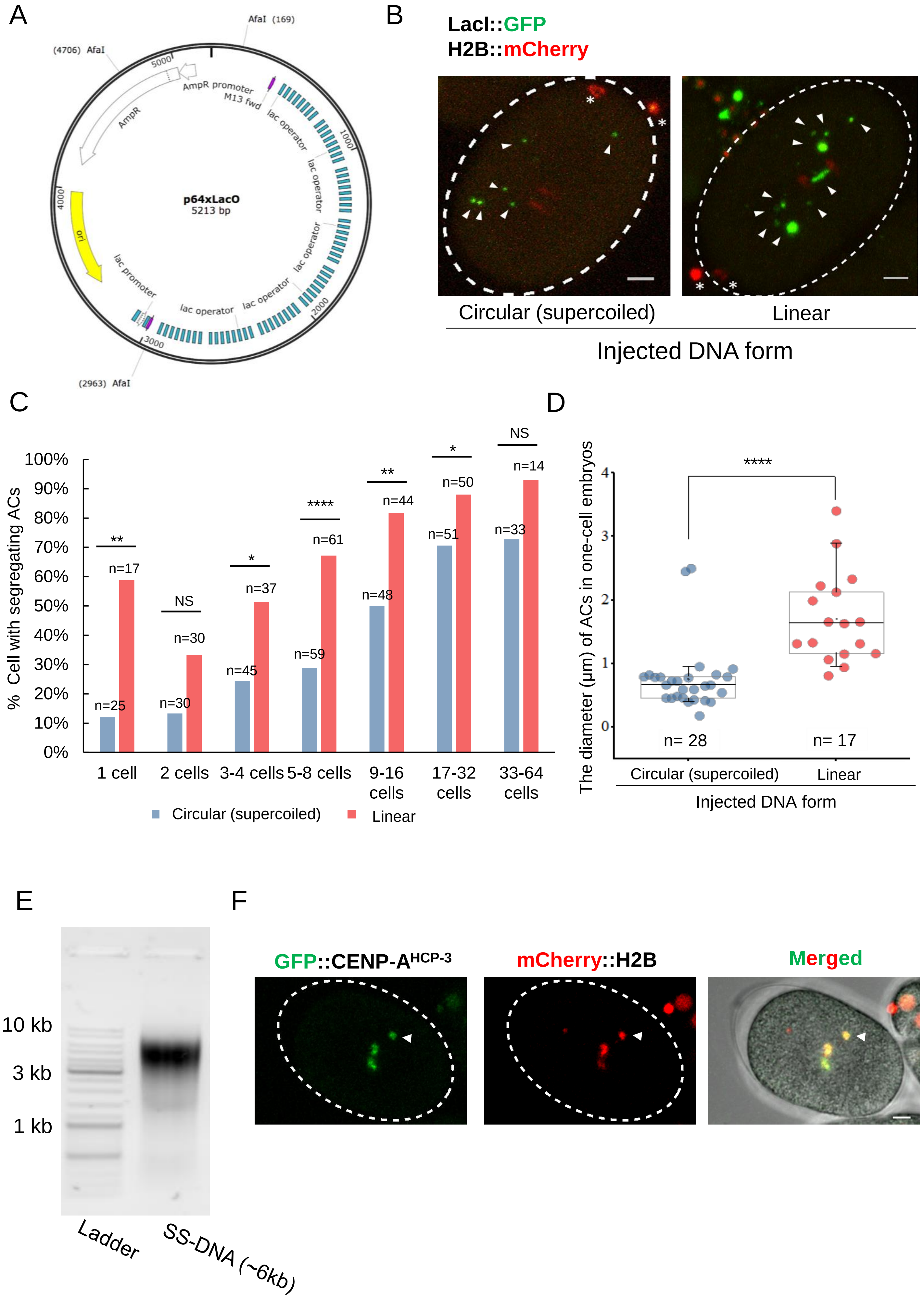

A
AfaI (169)
(4706) AfaI
5000
AmpR promoter
M13 fwd
AmpR
lac operator
1000
lac operator
4000
p64xLacO
5213 bp
ori
lac operator
lac promoter
lac operator
lac operator
lac operator
2000
3000
(2963) AfaI
B
LacI::GFP
H2B::mCherry
Circular (supercoiled)
Linear
Injected DNA form
C
% Cell with segregating ACs
100%
90%
80%
70%
60%
50%
40%
30%
20%
10%
0%
**
NS
*
****
**
*
NS
n=25
n=17
n=30
n=30
n=45
n=37
n=59
n=61
n=48
n=44
n=51
n=50
n=33
n=14
1 cell
2 cells
3-4 cells
5-8 cells
9-16 cells
17-32 cells
33-64 cells
Circular (supercoiled)
Linear
D
The diameter (μm) of ACs in one-cell embryos
****
4
3
2
1
0
n= 28
n= 17
Circular (supercoiled)
Linear
Injected DNA form
E
10 kb
3 kb
1 kb
Ladder
SS-DNA (~6kb)
F
GFP::CENP-A HCP-3
mCherry::H2B
Merged

Figure S1

G

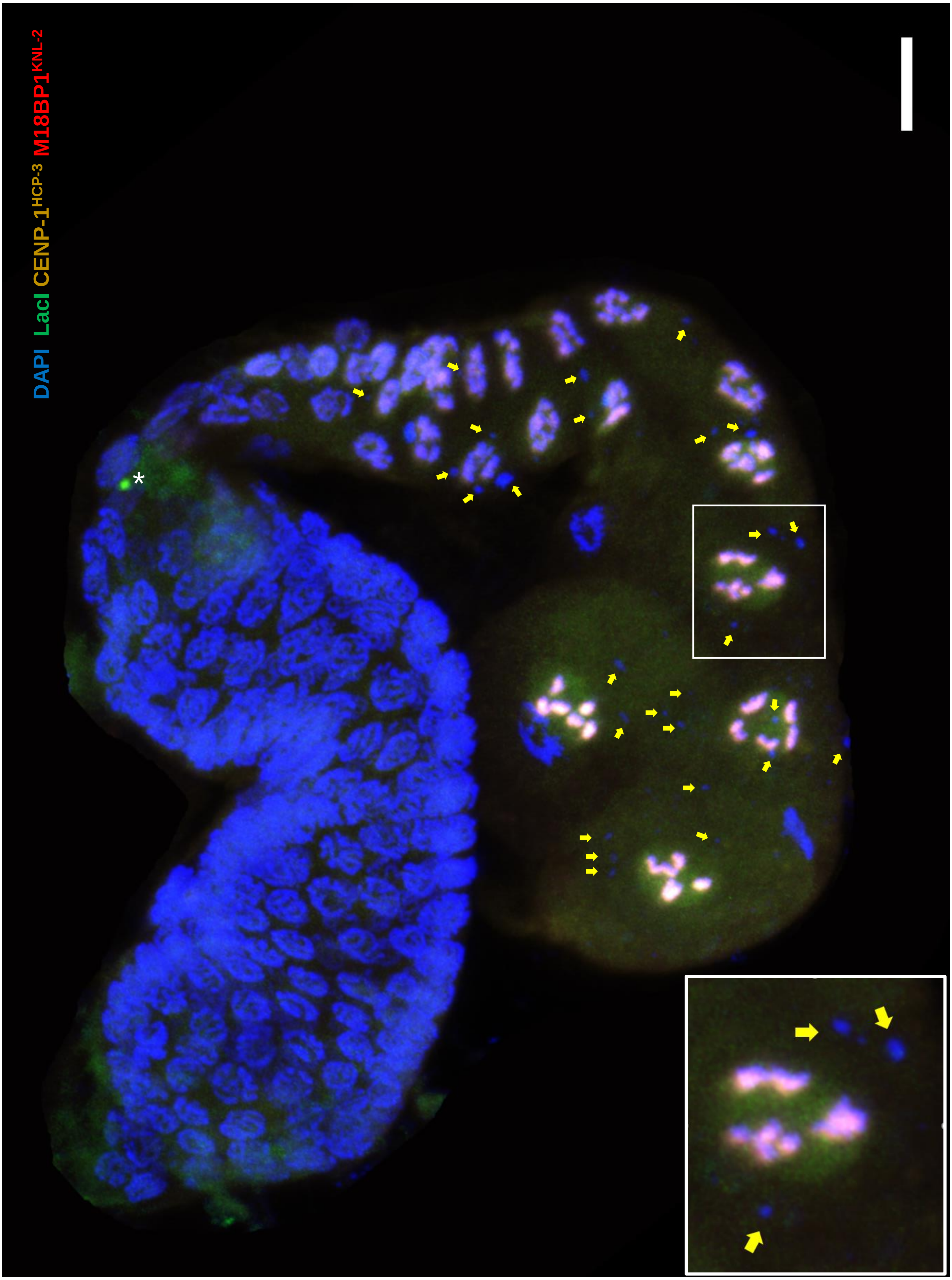

H

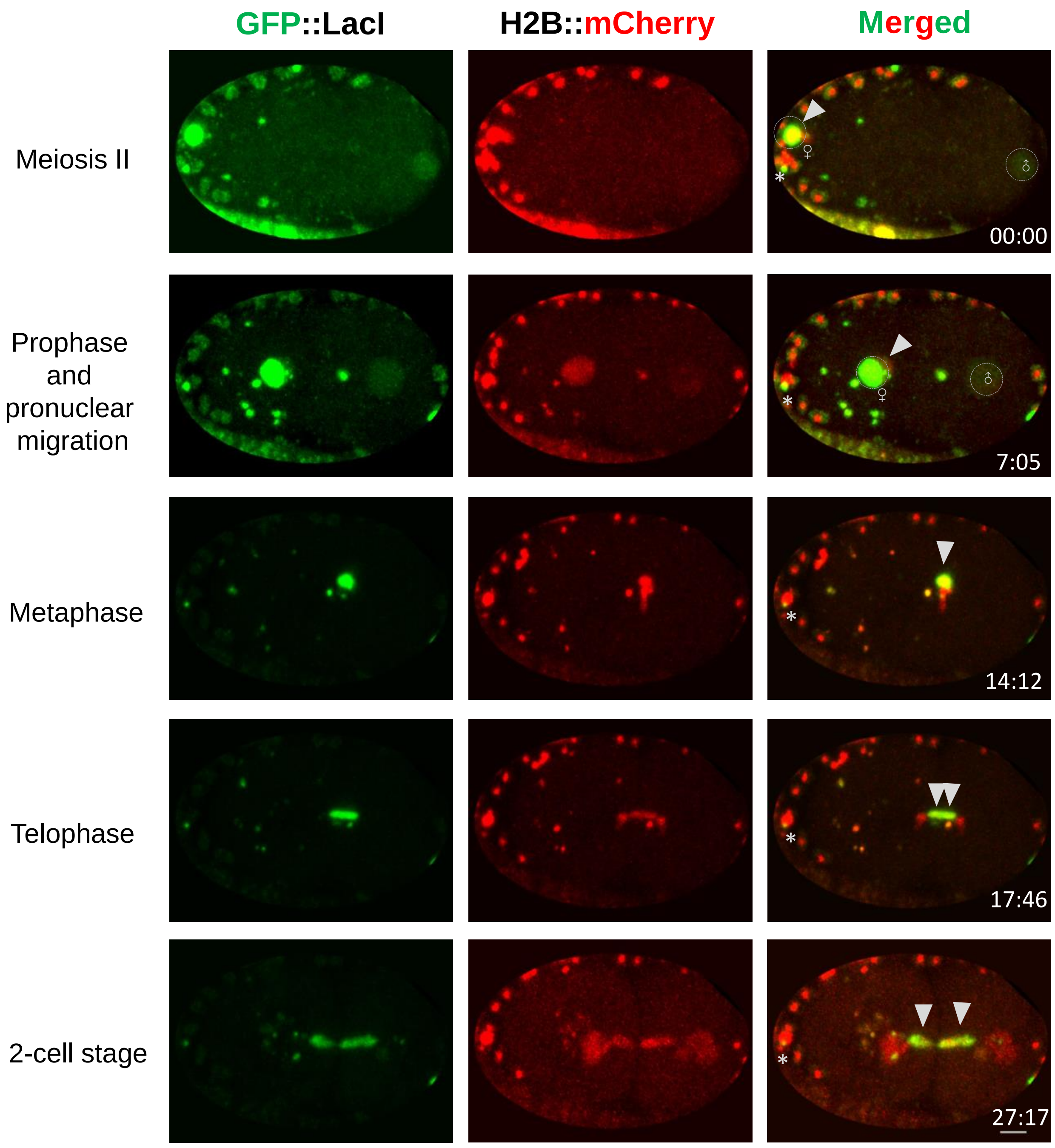
GFP::LacI
H2B::mCherry
Merged
Meiosis II
Prophase and pronuclear migration
Metaphase
Telophase
2-cell stage
00:00
7:05
14:12
17:46
27:17

I

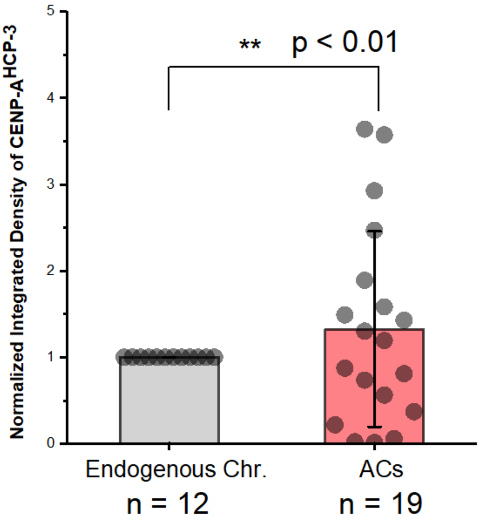
Normalized Integrated Density of CENP-A^{HCP-3}
** p < 0.01
Endogenous Chr.
n = 12
ACs
n = 19
0
1
2
3
4
5

Figure S2

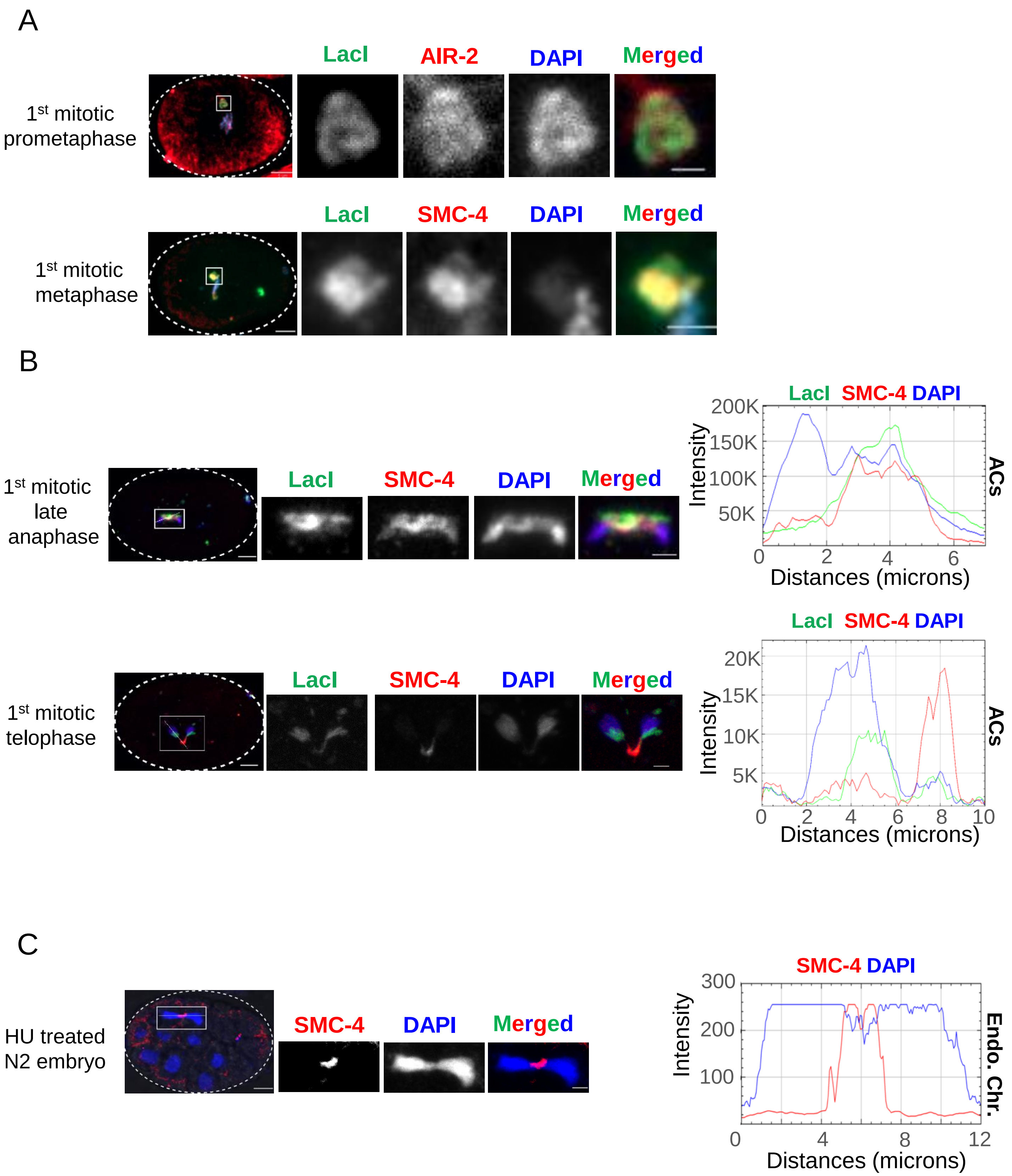
A
LacI
AIR-2
DAPI
Merged
1st mitotic prometaphase
LacI
SMC-4
DAPI
Merged
1st mitotic metaphase
B
1st mitotic late anaphase
LacI
SMC-4
DAPI
Merged
LacI SMC-4 DAPI
200K
150K
100K
50K
Intensity
0
2
4
6
Distances (microns)
ACs
1st mitotic telophase
LacI
SMC-4
DAPI
Merged
LacI SMC-4 DAPI
20K
15K
10K
5K
Intensity
0
2
4
6
8
10
Distances (microns)
ACs
C
HU treated N2 embryo
SMC-4
DAPI
Merged
SMC-4 DAPI
300
200
100
Intensity
0
4
8
12
Distances (microns)
Endo. Chr.

Figure S2

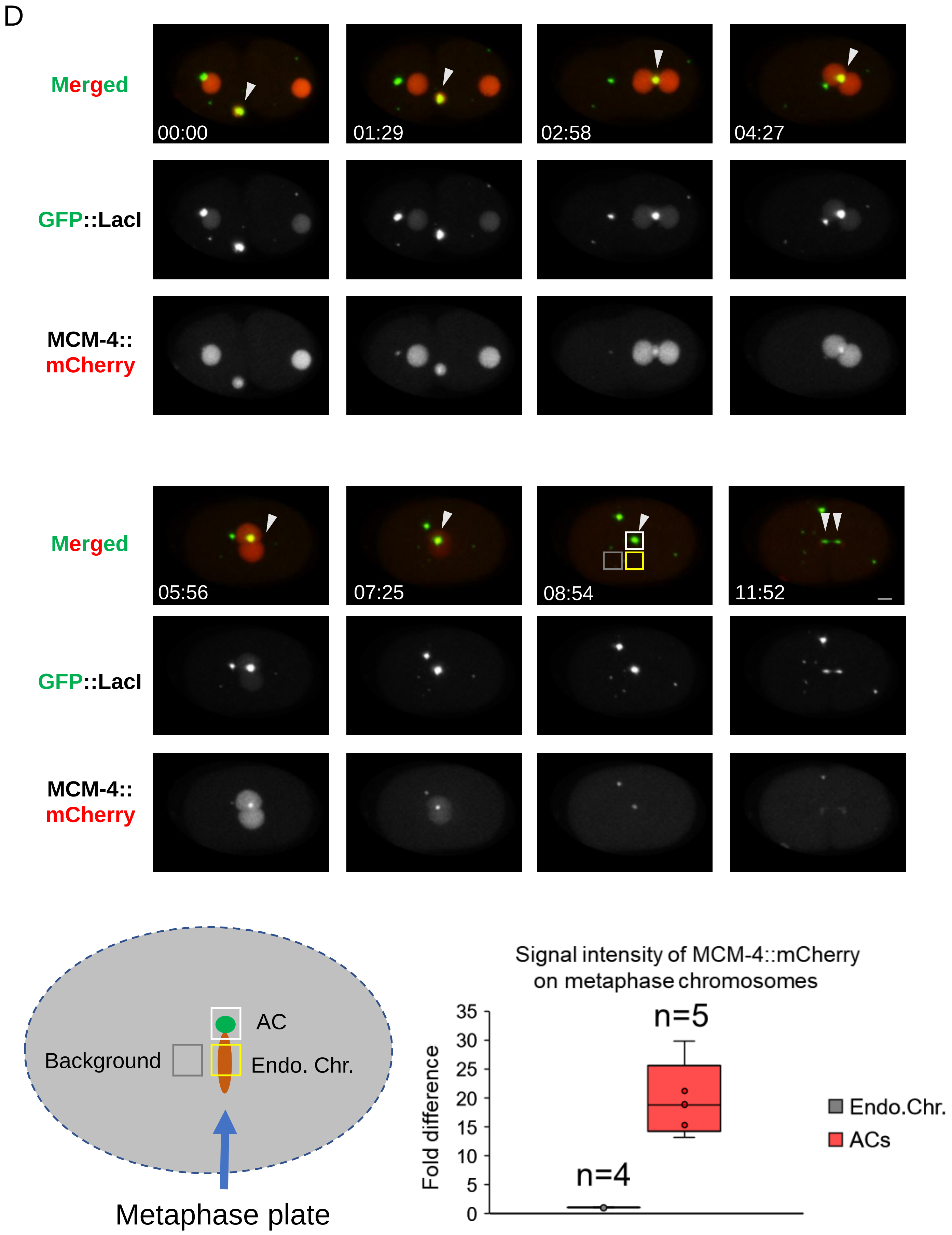
D
Merged
00:00
01:29
02:58
04:27
GFP::LacI
MCM-4::
mCherry
Merged
05:56
07:25
08:54
11:52
GFP::LacI
MCM-4::
mCherry
AC
Background
Endo. Chr.
Metaphase plate
Signal intensity of MCM-4::mCherry
on metaphase chromosomes
Fold difference
35
30
25
20
15
10
5
0
n=5
n=4
Endo.Chr.
ACs

Figure S2

E

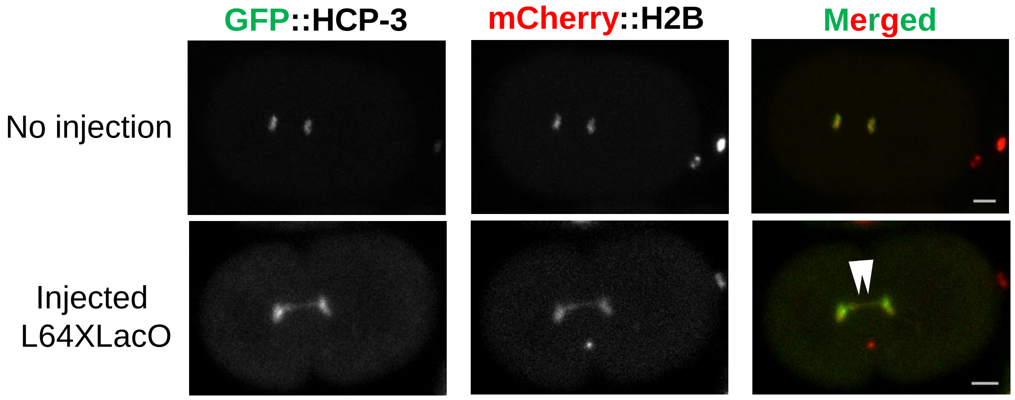

F

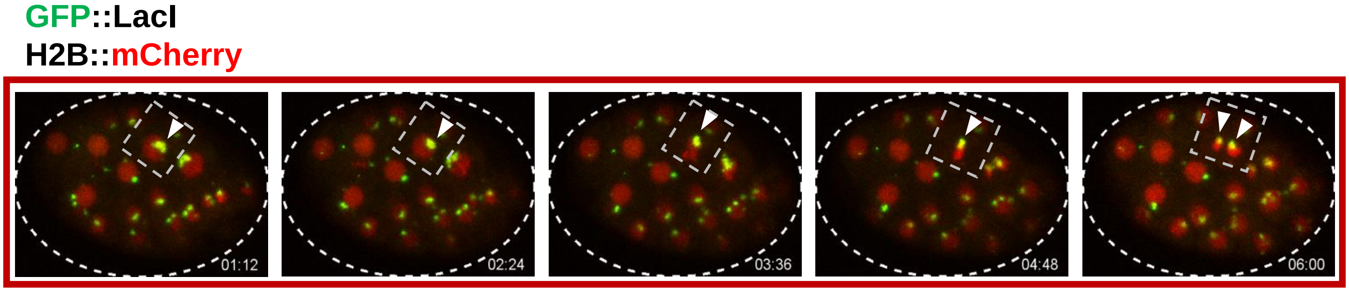

Proper AC segregation in multi-cell embryo

G

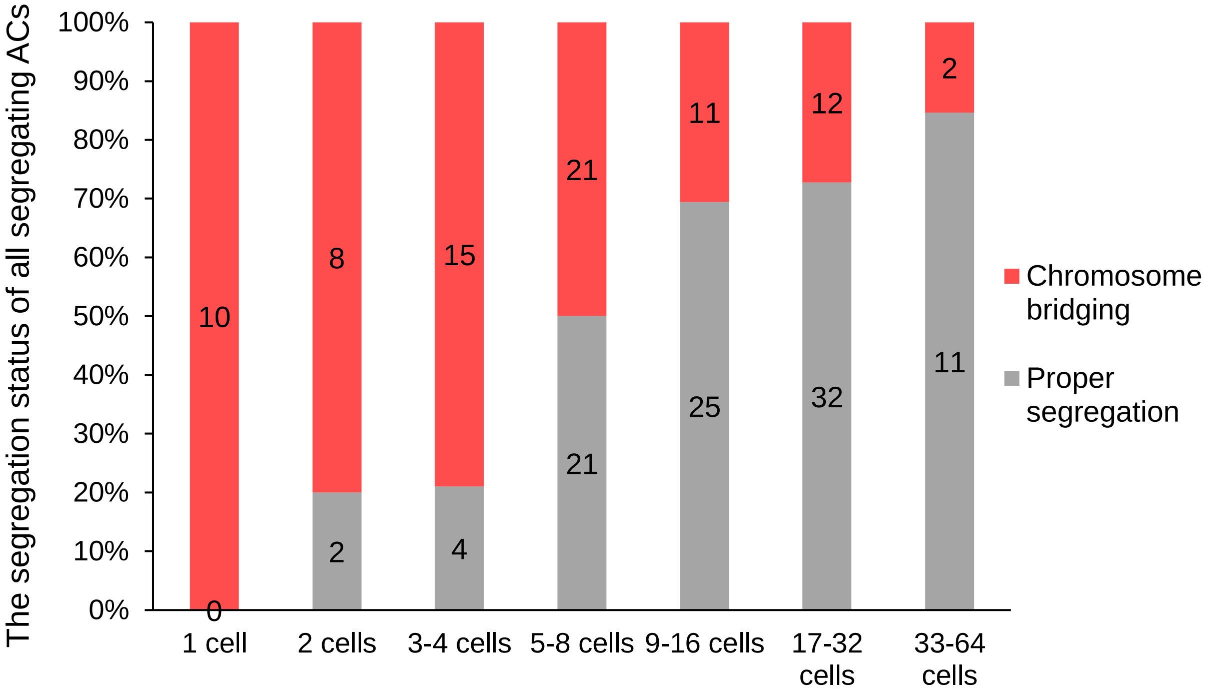

Figure S2

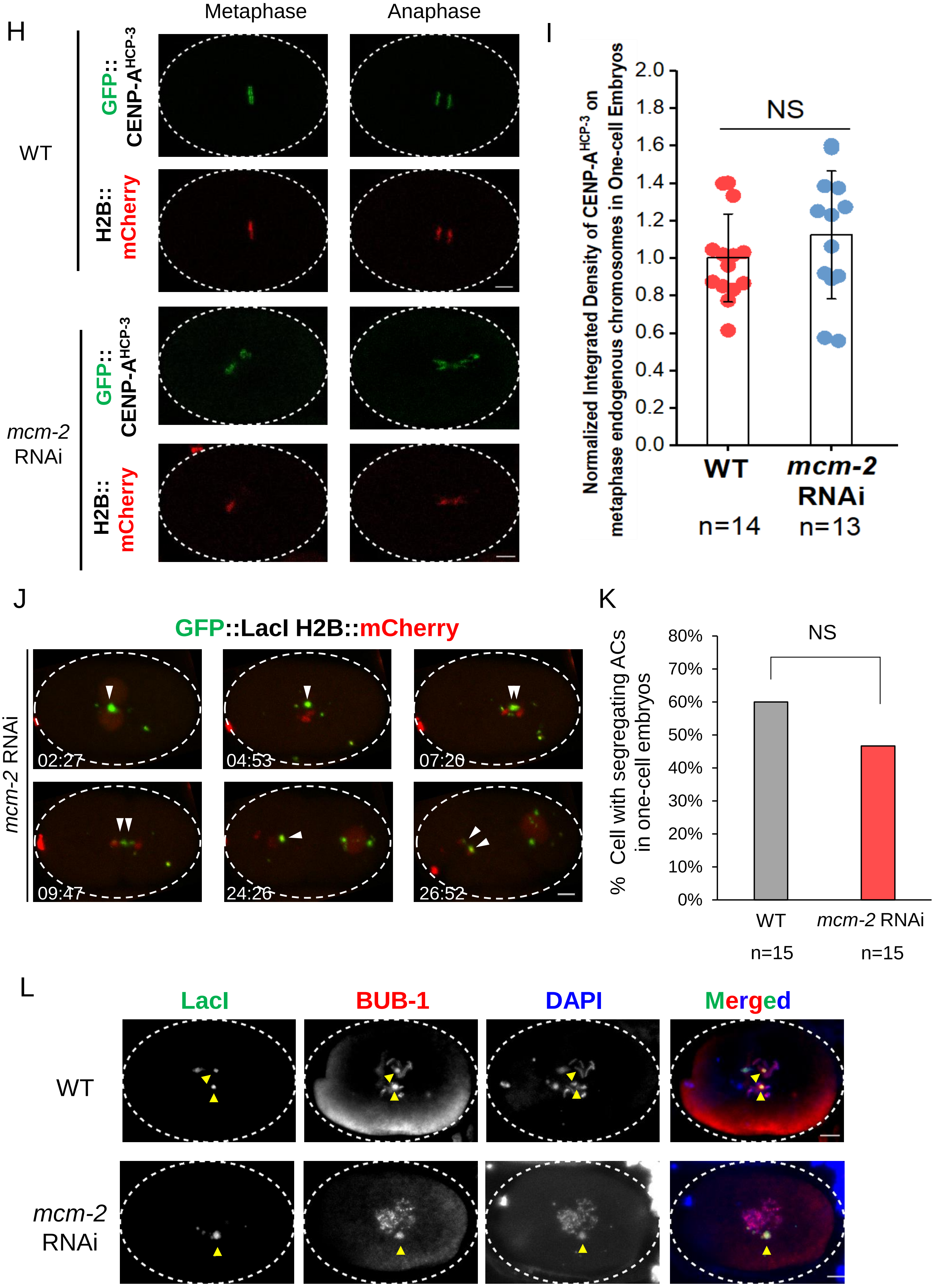

H
Metaphase
Anaphase
WT
GFP::CENP-A^HCP-3
H2B::mCherry
mcm-2 RNAi
I
NS
Normalized Integrated Density of CENP-A^HCP-3 on metaphase endogenous chromosomes in One-cell Embryos
2.0
1.8
1.6
1.4
1.2
1.0
0.8
0.6
0.4
0.2
0.0
WT
mcm-2 RNAi
n=14
n=13
J
GFP::LacI H2B::mCherry
mcm-2 RNAi
02:27
04:53
07:20
09:47
24:26
26:52
K
% Cell with segregating ACs in one-cell embryos
80%
70%
60%
50%
40%
30%
20%
10%
0%
NS
WT
mcm-2 RNAi
n=15
n=15
L
LacI
BUB-1
DAPI
Merged
WT
mcm-2 RNAi

Figure S3

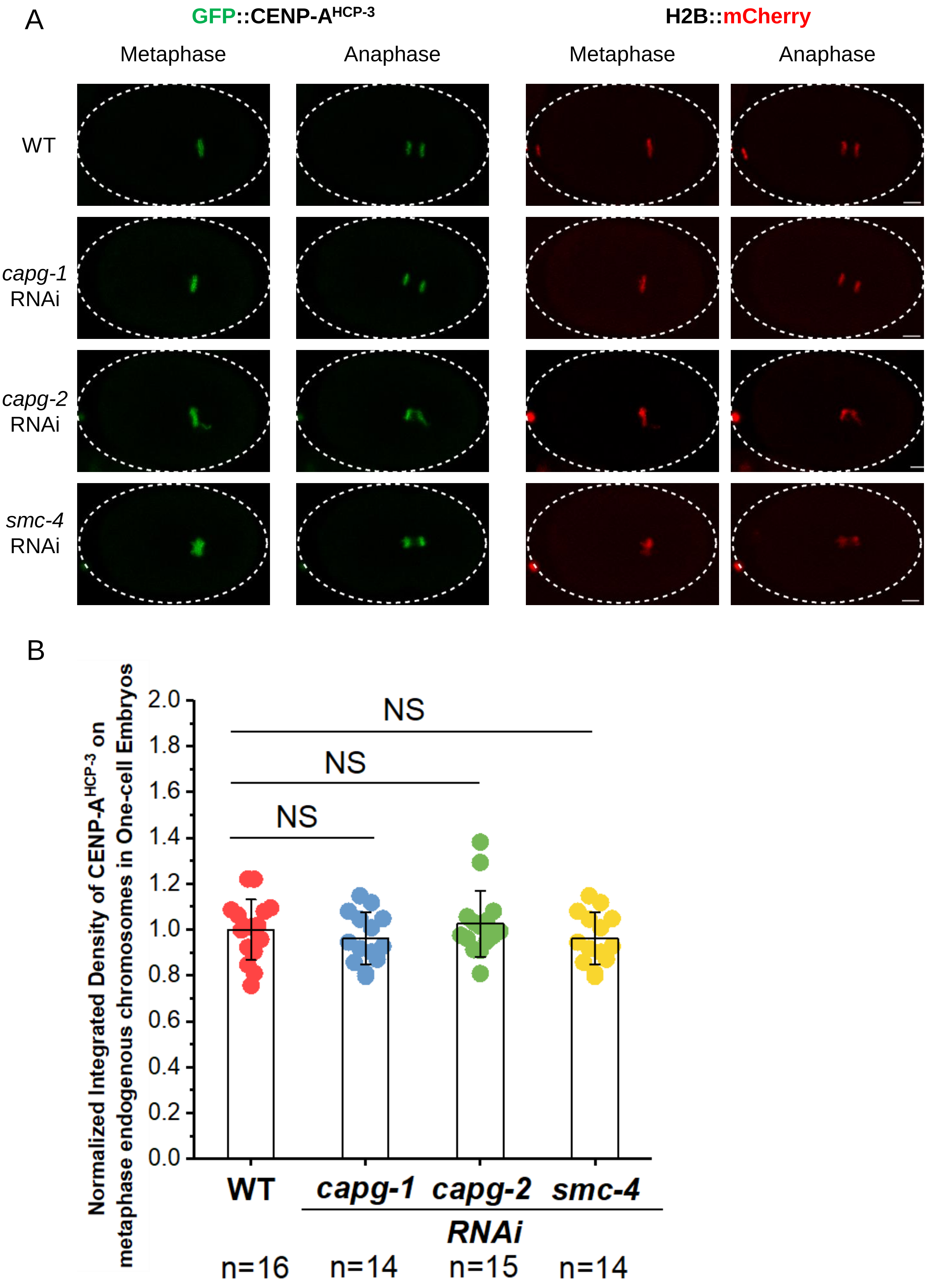
A
GFP::CENP-A HCP-3
H2B::mCherry
Metaphase
Anaphase
Metaphase
Anaphase
WT
capg-1 RNAi
capg-2 RNAi
smc-4 RNAi
B
Normalized Integrated Density of CENP-A HCP-3 on metaphase endogenous chromosomes in One-cell Embryos
2.0
1.8
1.6
1.4
1.2
1.0
0.8
0.6
0.4
0.2
0.0
NS
NS
NS
WT
capg-1
capg-2
smc-4
RNAi
n=16
n=14
n=15
n=14

Figure S4

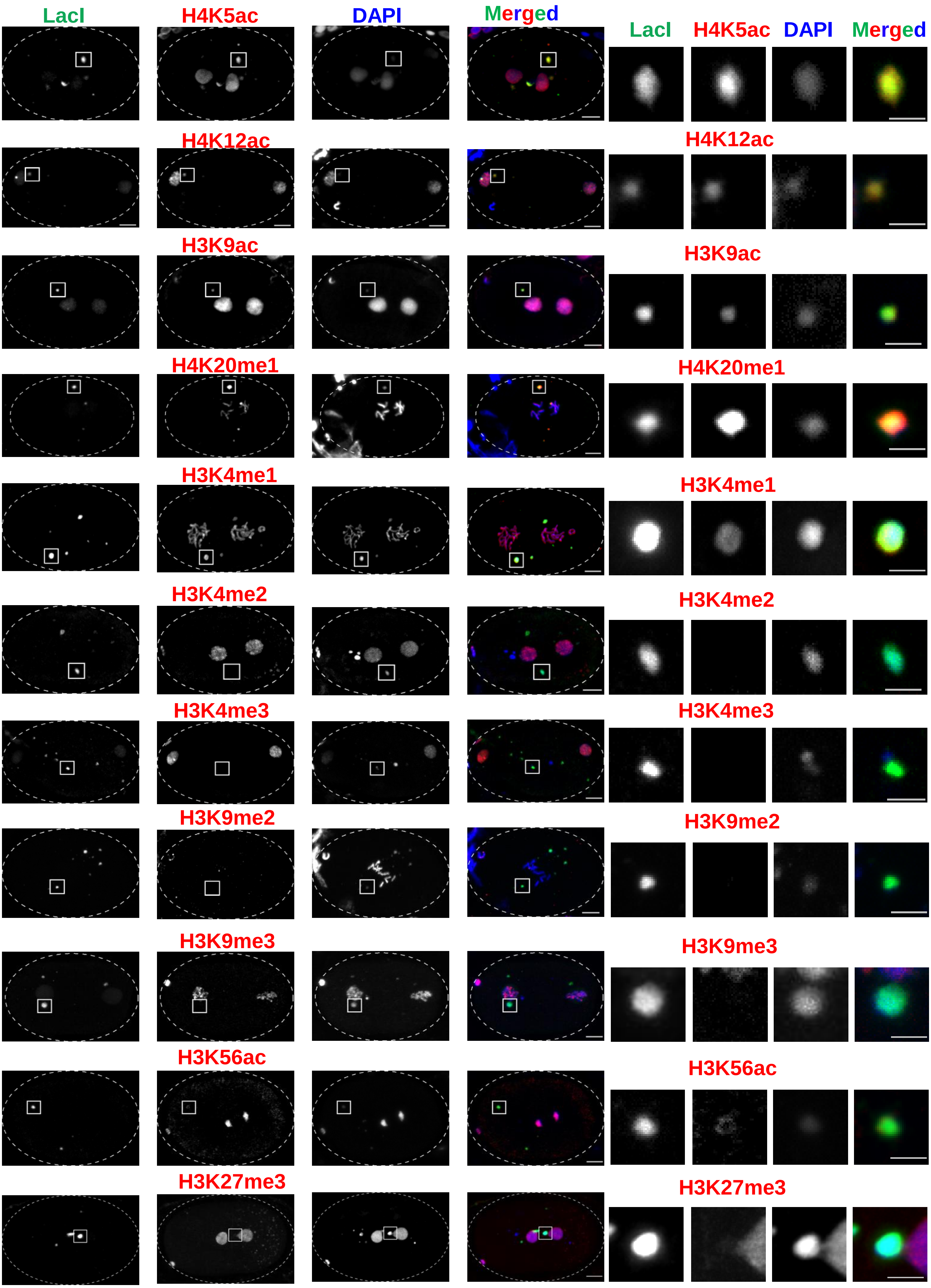
LacI
H4K5ac
DAPI
Merged
LacI
H4K5ac
DAPI
Merged
H4K12ac
H4K12ac
H3K9ac
H3K9ac
H4K20me1
H4K20me1
H3K4me1
H3K4me1
H3K4me2
H3K4me2
H3K4me3
H3K4me3
H3K9me2
H3K9me2
H3K9me3
H3K9me3
H3K56ac
H3K56ac
H3K27me3
H3K27me3

Figure S5

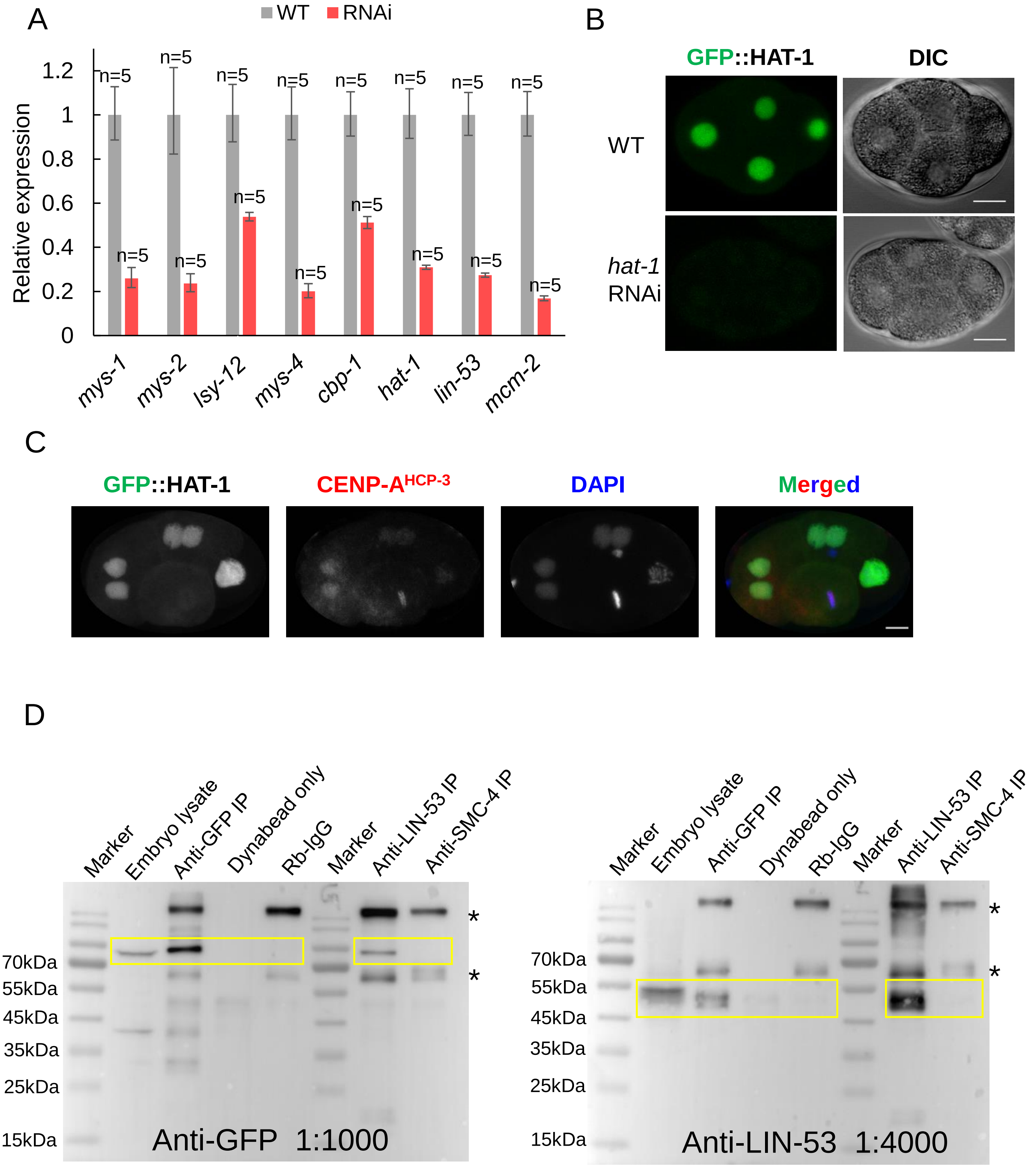
A
WT
RNAi
Relative expression
1.2
1
0.8
0.6
0.4
0.2
0
n=5
mys-1
mys-2
lsy-12
mys-4
cbp-1
hat-1
lin-53
mcm-2
B
GFP::HAT-1
DIC
WT
hat-1 RNAi
C
GFP::HAT-1
CENP-A HCP-3
DAPI
Merged
D
Marker
Embryo lysate
Anti-GFP IP
Dynabead only
Rb-IgG
Anti-LIN-53 IP
Anti-SMC-4 IP
70kDa
55kDa
45kDa
35kDa
25kDa
15kDa
Anti-GFP 1:1000
Anti-LIN-53 1:4000
*

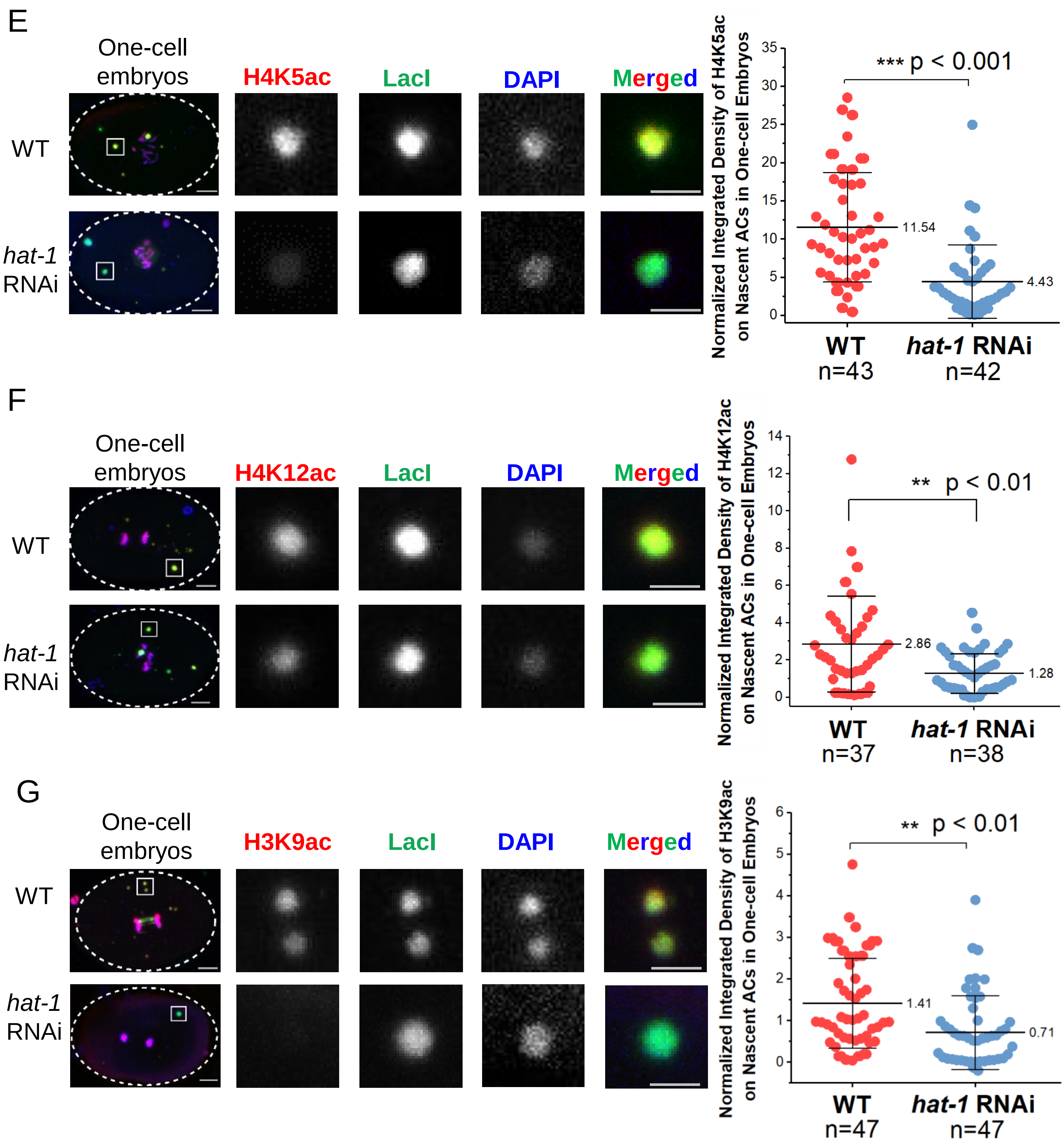
E
One-cell embryos
H4K5ac
LacI
DAPI
Merged
WT
hat-1 RNAi
Normalized Integrated Density of H4K5ac on Nascent ACs in One-cell Embryos
35
30
25
20
15
10
5
0
*** p < 0.001
11.54
4.43
WT
n=43
hat-1 RNAi
n=42
F
One-cell embryos
H4K12ac
LacI
DAPI
Merged
WT
hat-1 RNAi
Normalized Integrated Density of H4K12ac on Nascent ACs in One-cell Embryos
14
12
10
8
6
4
2
0
** p < 0.01
2.86
1.28
WT
n=37
hat-1 RNAi
n=38
G
One-cell embryos
H3K9ac
LacI
DAPI
Merged
WT
hat-1 RNAi
Normalized Integrated Density of H3K9ac on Nascent ACs in One-cell Embryos
6
5
4
3
2
1
0
** p < 0.01
1.41
0.71
WT
n=47
hat-1 RNAi
n=47

Figure S6

A

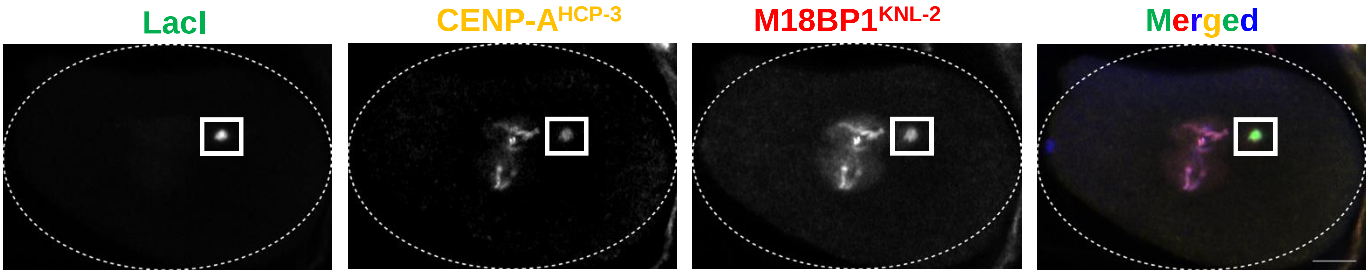
LacI
CENP-A^HCP-3
M18BP1^KNL-2
Merged

B

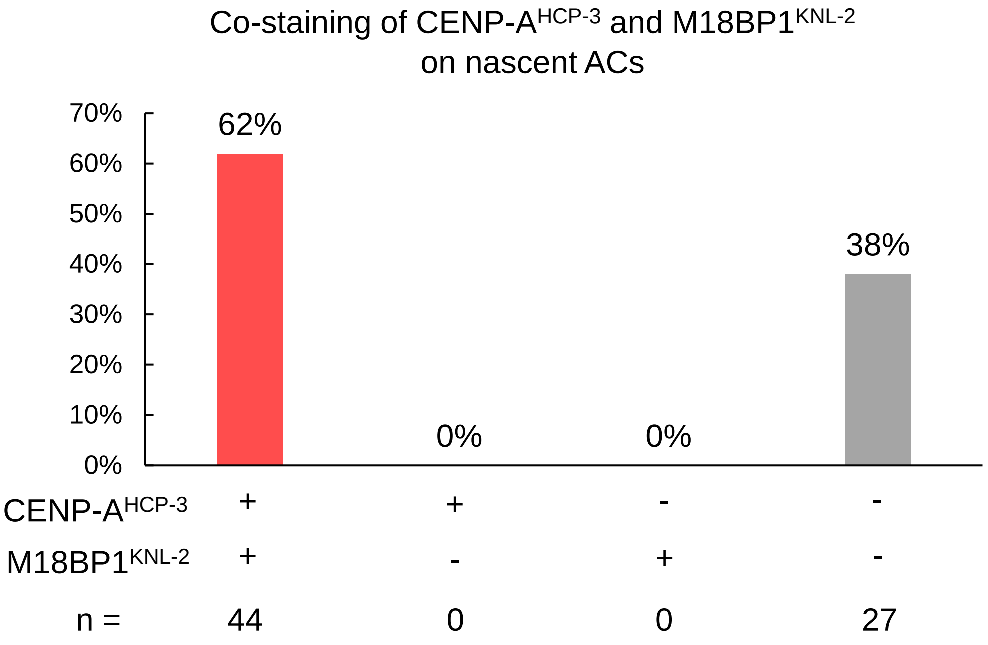
Co-staining of CENP-A^HCP-3 and M18BP1^KNL-2
on nascent ACs
70%
60%
50%
40%
30%
20%
10%
0%
62%
0%
0%
38%
CENP-A^HCP-3 + + - -
M18BP1^KNL-2 + - + -
n = 44 0 0 27
