# Supplementary material for: RbAp46/48LIN-53 and HAT-1 are required for initial CENP-AHCP-3 deposition and de novo holocentromere formation on artificial chromosomes in Caenorhabditis elegans embryos
Source: Nucleic Acids Res. 2021 Apr 19;49(16):9154–73. doi: 10.1093/nar/gkab217 (PMC8450102; doi:10.1093/nar/gkab217)
Supplement: gkab217_Supplemental_Files [file gkab217_supplemental_files.zip › LIN53AC_TableS1&S2_SupplementaryFigureLegends_20210306.pdf]

Table S1. *C. elegans* strans used in this study.

Strain name	Genotype	Construction Method	Reference
N2	Wild-type		
OD421	*unc-119 (ed3) III; ltSi4 [pOD833; hcp-3p/gfp::hcp-3; cb unc-119(+)] II; hcp-3(ok1892) III; ltIs37[pAA64; pie-1p/mCherry::his-58; unc-119 (+)]*		(1)
OD426	*ltIs37 [pie-1p::mCherry::his-58; unc-119(+)] IV; meIs1 [pie-1p::gfp::laci]*		(2)
WYY34	*meIs1 [pie-1p::GFP::LacI]; heSi45 [mcm-4::mCherry; unc-119(+)] IV*	OD426 males crossed with SV1067	This study
WYY44	*hat-1 (hku10 [gfp::hat-1]) III*	Cas9	This study
WYY46	*meIs1 [pie-1p::gfp::laci]*	OD426 males crossed with N2	This study
FAS46	*his-72 (uge30 [gfp::his-72]) III*	Cas9	(3)

Table S2. List of primers used for dsRNA production and genotyping.

Targeted Gene	Forward/ Reverse	Sequence (5' to 3')	Reference
hcp-3	Forward	GCAAAATGAGAGCGTCACAA	(4)
	Reverse	TCAGAGATGTCGAAGGCAGA	
knl-2	Forward	TCGACTTGGTCGGACAGATT	(4)
	Reverse	TGCGATATGTGGCGTTATGT	
lin-53	Forward	CCCCGTTCTTGTACGATCTC	(4)
	Reverse	GGCAGATTGGTCTTCTCCAA	
cbp-1	Forward	ACAAAATCAACCATGGGGAGGTGCT	(5)
	Reverse	ACACTGTATCTTCAGTTGGTGGCGG	
hat-1	Forward	AGATGGAAGTCTG TGG CCA ACG TG	(5)
	Reverse	GGCGCAAAATTTCGCGTATGGGTAA	
mys-1	Forward	GCTTGCCAAACTTTTTCTGG	(5)
	Reverse	TTCTTTTTCGCAGCCTCAAT	
mys-2	Forward	CCCCAATTGAGCAGAAGCCTGGTAT	(5)
	Reverse	AAATCGATAAAAACAGAGGCGGCGG	
lsy-12	Forward	CCAAATCCTTCGGTCGAAA	This study
	Reverse	TTACCGTTATCGGAACCTCG	
mys-4	Forward	CCACGATATGCCAAAATTCC	This study
	Reverse	TTTTGAATTCCCAGAATCCG	
set-1	Forward	GATTTCCACCACCTGGAATGTTTAG	This study
	Reverse	GCATCTCTCTGCCCGCTTC	
mcm-2	Forward	TGGCTGATCGTGCTAACAAC	This study
	Reverse	ACGTTCAACTCGAATGGTCC	
smc-4	Forward	GGAAAACATTTGGTTGCCGTT	This study
	Reverse	AAACGGAAGAGTCGAGGGAT	
capg-1	Forward	CATGCACCAACAGATCAACCTG	This study
	Reverse	TTGTAACCATTTCATGGCGATTTTCAGTC	
capg-2	Forward	GGATGTCTTTACCGAGCGCT	This study
	Reverse	CCAGTCTTGCGAGAACATCATTAG	
Seq-*hat-1*	Forward	TTTGCGATGAAAAATCTCACAACGAG	This study
	Reverse	CAAATTTACAGCAAATTTAGACGAATTTCGTG	

Figure S1. Deciphering how specific DNA structures used for microinjection affect the efficiency of new centromere formation. (A) The schematic plasmid map of p64xLacO for microinjection. To isolate circular, supercoiled DNA, p64xLacO was treated with T5 exonuclease to degrade linear ssDNA, linear dsDNA and nicked plasmid DNA. For linearization, p64xLacO was digested by *AfaI* to generate L64xLacO (2,794 bp). (B) Representative images of nascent ACs in one-cell embryos, which were generated from microinjection of either supercoiled DNA (left) or linear DNA (right). White arrowheads indicate the ACs. * represents polar bodies. Scale bar represents 5 µm. (C) Quantification of the segregation rate of ACs formed by injecting supercoiled DNA and linear DNA in 1-, 2-, 3-4-, 5-8-, 9-16-, 17-32-, 33-64-cell embryos. AC segregation rates were scored as the % of cells with segregating ACs among all dividing cells containing ACs. ACs formed by supercoiled DNA have lower segregation competency than linear DNA at each cell stage. Although the segregation competency of all types of ACs improves over time, the difference is especially clear in early embryo stages. The number of cells (n) analyzed was indicated. A chi-square test was used to test significance. *$p < 0.05$, **$p < 0.01$ and ****$p < 0.0001$. NS means not significant. (Same data set as from Lin and Yuen, submitted back-to-back. Figure S1D) (D) Quantification of the diameter of ACs (based on LacI::GFP dots) generated from injecting supercoiled DNA and linear DNA in one-cell embryos. The numbers of ACs (n) analyzed are shown. In the box plots, the boundary of the box closer to zero indicates the 25th percentile, a line within the box marks the median, a black dot within the box marks the mean, and the boundary of the box farther from zero indicates the 75th percentile. Whiskers above and below the box indicate the 10th and 90th percentiles. Student's t-test was used to test the significance. ****: $p < 0.0001$. (E) The agarose gel image of sheared salmon sperm DNA (SS-DNA), with a mean size of about 6 kbs, was used for microinjection. (F) ACs with “complex” DNA context formed from microinjection of SS-DNA show positive CENP-A^{HCP-3} and mCherry::H2B signals on the additional chromatin mass in a 1-cell embryo. The white arrowhead indicates the position of the AC. Scale bar represents 5 µm. (G) HMW DNA arrays formed in the gonad after microinjection. The maximum projection of the entire gonad shows that HMW DNA arrays localize in the diplotene and diakinesis region. * indicates an artefate green dot outside the gonad tissue. Scale bar represents 5 µm. (H) Seperated channels of time-lapse images following a segregating AC in first mitosis in Figure 1. Scale bar represents 5 µm. (I) The box plot shows the quantification of the integrated density of GFP::CENP-A^{HCP-3} signal on ACs in one-cell embryos (normalized to the endogenous chromosomes in the same embryo, which is set as 1).

Figure S2 (A) The inner centromeric protein, AIR-2, and the condensin II subunit, SMC-4, were both present on prometaphase ACs that were aligned on the metaphase plate. Immunofluorescence signal of SMC-4 persisted on (B) lagging ACs and on (C) the lagging endogenous chromosomes in a Hydroxyurea (HU)-treated embryo. Scale bar represents 5 µm. A higher-magnification view of the ACs (white square) is shown on the right, in which scale bar represents 2 µm. Lines were drawn across the lagging chromosomes, and the signal intensities were measured. The plot profiles show signal intensities from each channel, Green: LacI; Red: SMC-4; Blue: DNA. (D) Time-lapse images following

an nascent AC in one-cell embryo that expresses GFP::LacI (green) and MCM-4::mCherry (red) from interphase to anaphase. The fold difference of MCM-4::mCherry signal intensity on ACs and endogenous chromosomes is shown. The ACs that aligned at metaphase plates (white square) were used for calculating MCM-4::mCherry signal intensity. The yellow square represents the position of endogenous chromosomes, where MCM-4::mCherry is invisible. The signal intensity of MCM-4::mCherry on ACs and endogenous chromosomes were both subject to subtraction of the background signal (grey square). MCM-4::mCherry singanl intensity on ACs is about 20 fold higher than on endogenous chromosomes at metaphase, suggesting that DNA replication may still be ongoing on ACs. (E) An embryo expressing CENP-A^{HCP-3}::GFP and mCherry::H2B (without LacI::GFP) was subject to microjection of L64xLacO, and showed the phenomenon of lagging chromatin (white arrowhead) during anaphase. This suggests that AC lagging at anaphase is not caused by LacI::GFP tethering. (F) A representative image of a live cell (dashed line square) with an AC (white arrowhead), which segregated evenly in a multi-cell stage embryo. (G) The percentage of proper, equal AC segregation among all ACs that were attempting to segregate in different embryonic stages in WT. (H) GFP::CENP-A^{HCP-3} and mCherry::H2B localization on metaphase and anaphase chromosomes (endogenous) in one-cell embryos by live-cell imaging following untreated (WT) and *mcm-2* RNAi. Scale bar represents 5 μm. (I) Quantification of GFP::CENP-A^{HCP-3} signal intensity on mitotic chromosomes in *mcm-2* RNAi relative to WT. The number of embryos (n) analyzed is indicated. The integrated density of GFP::CENP-A^{HCP-3} was normalized to mCherry::H2B. Error bar indicates SD. Significant differences are analyzed by the student's t-test (NS, no significant). (J) Representative live-cell image of ACs attempting to segregate in *mcm-2* RNAi-treated one-cell embryos. The time lapses between the images are shown (mm:ss). Scale bar represents 5 μm. (K) Quantification of AC segregation rates in WT and *mcm-2* RNAi-treated one-cell embryos. The number of samples (n) analyzed is indicated. No significant differences are found between WT and *mcm-2* RNAi-treated one-cell embryos, as analyzed by Fisher's exact test (NS, $p > 0.05$). (L) Immunofluorescence of BUB-1 on ACs in wild-type (WT) or *mcm-2* RNAi-treated one-cell stage embryos at prometaphase. Scale bar represents 5 μm.

Figure S3. Condensin depletion does not affect CENP-A^{HCP-3} loading on endogenous chromosomes. (A) GFP::CENP-A^{HCP-3} and mCherry::H2B localization on metaphase and anaphase chromosomes (endogenous) in one-cell embryos by live-cell imaging following untreated (WT), *capg-1, capg-2* and *smc-4* RNAi. Scale bar represents 5 μm. (B) Quantification of GFP::CENP-A^{HCP-3} signal intensity on mitotic chromosomes in *capg-1, capg-2* and *smc-4* RNAi relative to WT. The number of embryos (n) analyzed is indicated. The integrated density of GFP::CENP-A^{HCP-3} was normalized to mCherry::H2B. Error bar indicates SD. Significant differences are analyzed by the student's t-test (NS, no significant).

Figure S4. Representative immunofluorescence images of H4K5ac, H4K12ac, H3K9ac, H4K20me, H3K4me, H3K4me2, H3K4me3, H3K56ac, H3K9me2, H3K9me3 and H3K27me3 on endogenous chromosomes and nascent ACs in one-cell embryos, with separated channels. Scale bar represents 5 μm. Scale bar represents 2 μm for the magnified images of ACs.

Figure S5. (A) RT-qPCR is used to confirm the RNAi efficiency after microinjection of dsRNA of

genes. (B) The expression of GFP::HAT-1 (WYY44) driven by the endogenous promoter in 4-cell embryos was observed by live-cell imaging with no treatment and *hat-1* RNAi treatment. Scale bar represents 10 μm. (C) GFP::HAT-1 does not localize on metaphase chromosomes. Scale bar represents 5 μm. (D) Co-immunoprecipitation of GFP::HAT-1, RbAp46/48$^{LIN\text{-}53}$ and SMC-4 using embryo extracts from the transgenic strain expressing GFP::HAT-1. No antibody (Dynabead only) and Rabbit IgG immunoprecipitation (IP) was used as negative controls. Inputs and immunoprecipitated proteins were analyzed by Western blots. Scatter plots shows the quantification of the normalized integrated density of (E) H4K5ac, (F) H4K12ac or (G) H3K9ac signal on ACs in WT (untreated) and *hat-1* RNAi-treated one-cell embryos. The number of ACs (n) analyzed was indicated. The integrated density of H4K5ac or H4K12ac was normalized to DAPI. Error bar indicates SD. Significant differences are analyzed by the student's t-test (**, $p < 0.01$; ***, $p < 0.001$).

Figure S6. (A) Immunofluorescence analysis of co-localization of CENP-A$^{HCP\text{-}3}$ and M18BP1$^{KNL\text{-}2}$ on nascent ACs in one-cell embryos. Scale bar represents 5 μm. (B) The percentages of nascent ACs that have both CENP-A$^{HCP\text{-}3}$ and M18BP1$^{KNL\text{-}2}$, have CENP-A$^{HCP\text{-}3}$ only, have M18BP1$^{KNL\text{-}2}$ only, and have neither CENP-A$^{HCP\text{-}3}$ nor M18BP1$^{KNL\text{-}2}$ were shown. n represents the number of cells analyzed.

References

1. Gassmann, R., Rechtsteiner, A., Yuen, K.W., Muroyama, A., Egelhofer, T., Gaydos, L., Barron, F., Maddox, P., Essex, A., Monen, J. *et al.* (2012) An inverse relationship to germline transcription defines centromeric chromatin in C. elegans. *Nature*, **484**, 534-537.
2. Yuen, K.W., Nabeshima, K., Oegema, K. and Desai, A. (2011) Rapid de novo centromere formation occurs independently of heterochromatin protein 1 in C. elegans embryos. *Current biology : CB*, **21**, 1800-1807.
3. Delaney, K., Mailler, J., Wenda, J.M., Gabus, C. and Steiner, F.A. (2018) Differential Expression of Histone H3.3 Genes and Their Role in Modulating Temperature Stress Response in Caenorhabditis elegans. *Genetics*, **209**, 551-565.
4. Lee, B.C., Lin, Z. and Yuen, K.W. (2016) RbAp46/48(LIN-53) Is Required for Holocentromere Assembly in Caenorhabditis elegans. *Cell reports*, **14**, 1819-1828.
5. Ho, V.W., Wong, M.K., An, X., Guan, D., Shao, J., Ng, H.C., Ren, X., He, K., Liao, J., Ang, Y. *et al.* (2015) Systems-level quantification of division timing reveals a common genetic architecture controlling asynchrony and fate asymmetry. *Mol Syst Biol*, **11**, 814.
